# Evaluation of a custom QIAseq targeted DNA panel with 164 ancestry informative markers sequenced with the Illumina MiSeq

**DOI:** 10.1038/s41598-021-99933-2

**Published:** 2021-10-26

**Authors:** D. Truelsen, A. Freire-Aradas, M. Nazari, A. Aliferi, D. Ballard, C. Phillips, N. Morling, V. Pereira, C. Børsting

**Affiliations:** 1 Section of Forensic Genetics, Department of Forensic Medicine, Faculty of Health and Medical Sciences, University of Copenhagen, 2100 Copenhagen, Denmark; 2 Forensic Genetics Unit, Institute of Forensic Sciences, University of Santiago de Compostela, Santiago de Compostela, Spain; 3 Faculty of Life Sciences and Medicine, King’s College, London, UK; 4 Department of Mathematical Sciences, Aalborg University, 9220 Aalborg, Denmark

**Keywords:** Biological techniques, Genetics, Molecular biology

## Abstract

Introduction of new methods requires meticulous evaluation before they can be applied to forensic genetic case work. Here, a custom QIAseq Targeted DNA panel with 164 ancestry informative markers was assessed using the MiSeq sequencing platform. Concordance, sensitivity, and the capability for analysis of mixtures were tested. The assay gave reproducible and nearly concordant results with an input of 10 and 2 ng DNA. Lower DNA input led to an increase in both locus and allele drop-outs, and a higher variation in heterozygote balance. Locus or allele drop-outs in the samples with less than 2 ng DNA input were not necessarily associated with the overall performance of a locus. Thus, the QIAseq assay will be difficult to implement in a forensic genetic setting where the sample material is often scarce and of poor quality. With equal or near equal mixture ratios, the mixture DNA profiles were easily identified by an increased number of imbalanced heterozygotes. For more skewed mixture ratios, the mixture DNA profiles were identified by an increased noise level. Lastly, individuals from Great Britain and the Middle East were investigated. The Middle Eastern individuals showed a greater affinity with South European populations compared to North European populations.

## Introduction

There is an increasing interest in sequencing and analysing ancestry informative markers (AIMs) in forensic genetic casework because AIMs may generate investigative leads in cold cases and support the identification of missing persons^1^. The introduction of massively parallel sequencing (MPS) simplified the typing of high numbers of single nucleotide polymorphisms (SNPs) that are typically used to infer the ancestry of an unknown individual^2,3^. Currently, there are two commercial MPS panels available for AIM SNP genotyping: the Precision ID Ancestry Panel^4–7^ and the ForenSeq DNA Signature Prep Kit^7–12^. Both panels amplify AIMs that are useful for the continental differentiation of human population groups. With these and other well-established panels in place, the search for markers that may resolve genetic patterns on a fine geographical scale has been initiated^13–15^. From a forensic perspective, the ability to differentiate individuals of Middle Eastern ancestry from individuals with European ancestry is challenging. The Middle East has throughout time been the centre of many different population migrations between Africa, Asia, and Europe, which have altered the demography and genetic structure within the area^16–18^. Large amounts of gene flow from the surrounding regions have made individuals from the Middle East particularly difficult to differentiate from Europeans and South Asians using global ancestry panels^13,19–22^.

Several PCR-based assays such as the Ion AmpliSeq (Thermo Fisher Scientific), GeneRead (Qiagen), and the ForenSeq DNA Signature Prep Kit (Verogen) have been used in forensic genetics for various purposes^4,10,23–27^. Here, we evaluated the applicability of the QIAseq chemistry for forensic genetic case work using a custom panel of 164 SNPs specifically selected to differentiate Middle Eastern, South Asian, and European populations^15,24^. The QIAseq chemistry was originally designed for cancer diagnostics to detect variants with low frequencies in cell populations^28,29^. The assay has been used in forensic genetics for the identification of mass disaster and genocide victims through the work by the International Commission on Missing Persons (https://www.icmp.int/). In the QIAseq assay, the first step of the protocol is an enzymatic fragmentation of the DNA followed by ligation of adapters with molecular barcodes known as unique molecular indices (UMI). UMIs increase the sensitivity of variant detection and allow identification of variants present in extremely low frequencies such as tumour-derived mutations^28^. True variants will be present in almost all reads with the same UMI, whereas PCR or sequencing errors are easily detected as artefacts since they are only present in some of the reads with the same UMI^30^. The QIAseq assay can be sequenced with both the MiSeq (Illumina) and the Ion S5 (Thermo Fisher Scientific). In this work, the test was performed with the MiSeq.

When evaluating new chemistries in a forensic context, it is important to investigate the adequacy of these methods to low and/or poor quality DNA samples and to define stringent quality standards for data analysis. With this in mind, we investigated; (1) the sensitivity of the assay, (2) the concordance with previously obtained results to assure correct sequencing of genotypes, (3) the reproducibility within and between labs, and (4) the capability to identify mixtures. Individuals from Syria, individuals self-declared as of Middle Eastern origin (primarily from Iran and Iraq), and individuals from Great Britain were genotyped. This was done to test the ability of the selected markers to differentiate Europeans from individuals from the Middle East. The aim was to evaluate if the QIAseq assay proved to be reliable and sensitive enough in a forensic context, and if it could be used as an alternative for library preparation to other assays such as the AmpliSeq or the GeneRead assays.

## Results and discussion

### Sensitivity

The sensitivity of the QIAseq assay was first assessed by sequencing DNA from a Danish individual with known genotypes using an input of 10 ng, 2.0 ng, 0.5 ng, 0.25 ng, and 0.125 ng genomic DNA. A control sample of 40 ng from the same individual was also genotyped at University of Copenhagen (UCPH) as the QIAseq protocol recommends an input of 10–40 ng DNA. At UCPH, the dilution series was typed in duplicate, and at King’s College, London (KCL) it was typed once.

**Figure 1:**
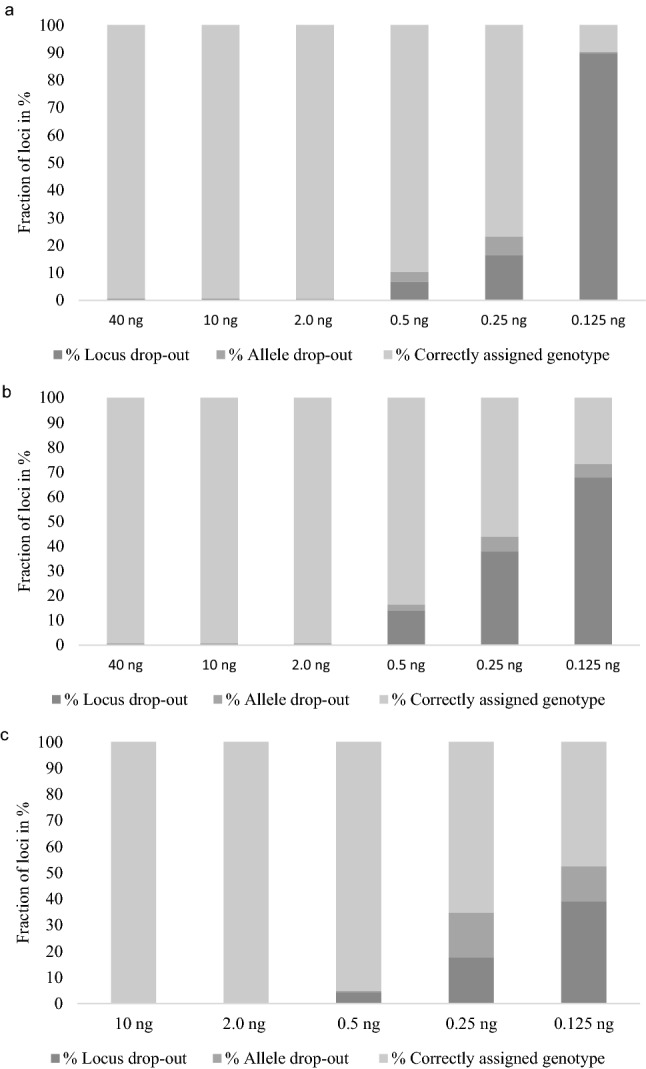
Sensitivity study. Percentage of correctly assigned genotypes, locus- and allele drop-outs for each dilution using a minimum read depth of 20 reads and a heterozygote balance of 0.3–3.0. (**a**) Samples analysed at UCPH, replicate 1; (**b**) samples analysed at UCPH, replicate 2; (**c**) samples analysed at KCL. Light grey indicates the % correctly assigned genotypes, medium grey indicates the % allele drop-outs, and dark grey indicates the % locus drop-outs.

Figure 1 shows the percentage of correctly assigned genotypes, locus- and allele drop-outs for each dilution with a minimum read depth of 20 and heterozygote balance (Hb) of 0.3–3.0. For both replicates of the control sample with 40 ng input DNA, the SNP rs718501 presented Hb above 3.0.

This particular locus was also problematic with 10 ng and 2 ng input DNA. A total of six discordances were found. Three discordances were due to locus drop-outs (Hb > 3.0), and the other three discordances were due to allele drop-outs. The known rs718501 genotype of the Danish individual was rs718501 [AC], whereas it was determined as either [AA] or [AC] with the QIAseq assay. The SNP rs718501 generally performed poorly with all dilutions tested in the sensitivity study (Supplementary Table S3). The locus either displayed Hb outside the specified threshold (4 dilutions), allele drop-outs (5 dilutions) or locus drop-out (4 dilutions). In contrast, the 50 Syrian individuals had a median Hb of 1.048, a median locus balance of 1.395, and a median noise of 0.703% for this particular locus. The discrepancies observed in the Danish sample was likely caused by poor amplification of the C allele (92% of all reads had the A allele) due to sequence variation in the target specific PCR primer binding site. Besides these eight inconsistencies for the same locus, full concordance was obtained with 40 ng, 10 ng, and 2 ng of DNA both between replicates and between laboratories. In comparison, forensic genetic laboratories routinely generate DNA profiles from 100 to 200 pg DNA using commercial or in-house developed assays^31–34^.

As expected, the number of drop-outs increased with decreasing amount of input DNA. However, there was no obvious pattern in the loci that suffered from drop-outs (Supplementary Tables S3–S5). The numbers of locus or allele drop-outs between replicates, laboratories, and DNA input amounts were not consistent (Supplementary Tables S4, S5). Locus drop-outs were not necessarily observed in loci with low read depths (Supplementary Fig. S1) and allele drop-outs were not necessarily observed in loci with skewed heterozygote balances (Supplementary Fig. S2) or high levels of noise (Supplementary Fig. S3).

The 96 allele drop-outs observed in the sensitivity study were divided among 46 different loci (Supplementary Table S5) and in 17 of these loci, allele drop-out was only observed once. These features make it difficult to select and exclude poorly performing loci from the assay. Overall, fewer locus drop-outs were observed at KCL that also had higher read depths compared to UCPH data. However, while the number of locus drop-outs was lower for KCL, the number of allele drop-outs was higher for analyses with 0.25 and 0.125 ng input DNA compared to the UCPH data. When 0.125 ng of input DNA was used, more than 50% of the genotype calls were incorrect for both KCL and UCPH (Fig. 1).

**Figure 2:**
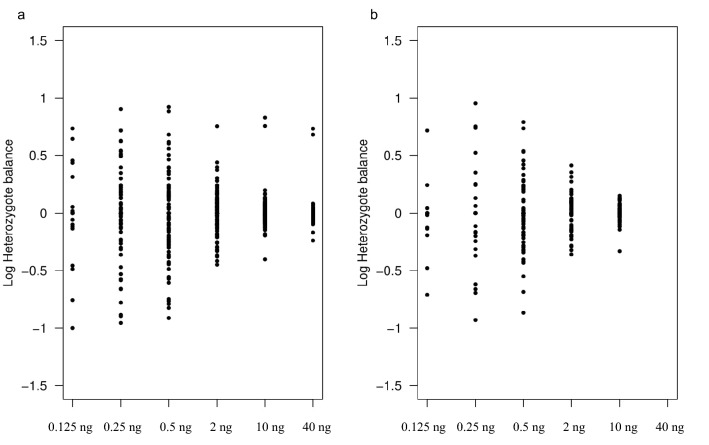
Log transformed heterozygote balances in the sensitivity study. (**a**) UCPH. (**b**) KCL. Heterozygote balance was estimated as the number of reads for a nucleotide divided by the number of reads for the other nucleotide in the called genotype in the following order: A, C, G, and T. The outliers observed for 40 and 10 ng DNA input (**a**) represent the rs718505 locus as discussed in the sensitivity paragraph.

Figure 2 shows the Hb for the different amounts of DNA used in the sensitivity study (minimum read depth = 20). The balance for genotypes ranged from 0.1 to 8.4 for the UCPH sensitivity study, and from 0.12 to 9.0 for the KCL sensitivity study. Increased Hb with decreasing DNA input was observed in both laboratories (Fig. 2a and 2b). A total of 10.3% and 9.5% of the heterozygous genotypes in the UCPH and KCL sensitivity studies were outside the acceptable Hb range. This was much higher than those observed with the GeneRead and AmpliSeq assays, where only 0.12% and 0.17% of the genotypes were outside the defined threshold of 0.3–3.0^23,24^. The reduced sensitivity and large variation in read depth observed for the QIAseq assay compared to the two PCR-MPS assays may be attributed to the different library preparation methods. The reduced sensitivity of the assay and the randomness in locus- and allele drop-outs indicated that the adapter ligation step was not efficient. Overall, the results showed that although the genotyping was reproducible for high input amounts of DNA, the QIAseq assay was not sufficiently sensitive to meet the requirements in forensic genetics.

### Genotype concordance

Four Coriell samples (two Central European (CEU) individuals, one individual from Yoruba, Ibadan, Nigeria (YRI), and one Southern Han Chinese individual from China (CHS)) were typed with the QIAseq assay, and genotype concordance was checked with known reference data. In total, three discordances were observed. Two discordances were due to locus drop-out in the QIAseq assay for rs1470637 for one CEU individual and the YRI individual. One discordance was an allele drop-out in rs4834738 using the QIAseq assay for one of the CEU individuals (NA06994). The reference data reported the genotype as [CT], while the QIAseq assay typed the individual as [TT]. This was observed by both UCPH and KCL. An analysis in IGV showed around 2.6% reads for the C allele. This could indicate the presence of a partial null allele in the CEU individual for this particular locus. The four Coriell samples were sequenced using 10 ng DNA and were therefore within the range specified in the QIAseq protocol. Besides the three discordances, concordant genotypes were obtained, which underlined that the QIAseq genotyping was reliable when sufficient amounts of DNA were used.

### Mixtures

**Figure 3:**
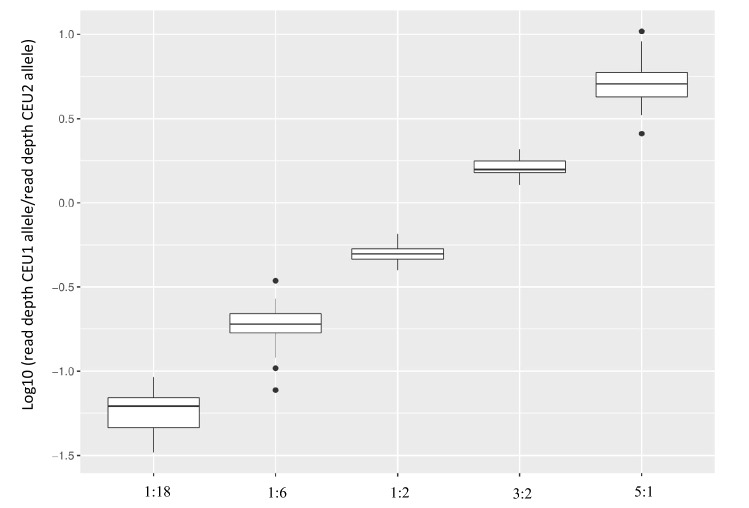
Mixture study. The read depth ratio for the nine loci for which the two individuals were opposite homozygotic. The read depth ratio was calculated as the number of reads for the allele of CEU1 divided by the number of reads for the allele of CEU2.

Samples with DNA contributions from two or more people are frequently collected from crime scenes, which makes it important to identify a given sample as a mixture. With bi-allelic loci such as SNPs and insertion/deletions, this requires a detailed analysis of the allele read count(s). DNA mixtures were constructed from DNA from the two CEU Coriell samples (CEU1 and CEU2) in ratios of 1:18, 1:6, 1:2, 3:2, and 5:1, and the ability to identify mixtures was evaluated. The two samples had different genotypes at 78 loci. At nine out of these 78 positions, the two individuals were opposite homozygotes. Figure 3 shows the ratio of allele counts for CEU1 divided by the allele counts for the CEU2 individual for the nine loci. Ratios were log10-transformed to allow better comparison. The ratio between the read counts for the two alleles at the nine loci were consistent with the ratio of the mixture.

For the mixtures of 1:2, 1:6, 3:2, and 5:1, the called genotypes at the nine opposite homozygote loci were mixtures of one allele from each contributing individual. For the 1:18 mixture, the called genotypes at the nine loci were those of the major contributor only (CEU2). However, the read depth of the alleles from the minor contributor was still above the read depth threshold of 20 reads. Table 1 shows the number of genotypes that fell outside the Hb threshold of 0.3–3.0. The number of imbalanced heterozygous genotypes increased markedly for the mixed DNA samples compared to those of the single source samples, which demonstrated that it was possible to identify mixtures by identifying an increased number of heterozygote genotypes with a high heterozygote imbalance. This pattern has been observed previously^32,35^.

**Table 1:** Mixture study.

Sample mixture ratio	No. of genotypes outside specified Hb thresholds*	Range of Hb	Number of loci with noise^†^ > 3%	Average number of observed heterozygotes	Average number of observed homozygotes
1:2	67	0.12–8.4	1	96.5	67.5
1:6	27	0.11–8.67	56	69	95
1:18	1	0.21–2.59	55	55	109
3:2	61	0.14–7.65	2	96	68
5:1	45	0.12–8.93	42	75.5	88.5

Single source samples	No. of genotypes outside specified Hb thresholds*	Range of Hb	Number of loci with noise^†^ > 3%	Average number of observed heterozygotes	Average number of observed homozygotes
CEU1	0	0.4–1.43	12	52	112
CEU2	1	0.38–3.40	0	55	109

*Heterozygote balance thresholds used: 0.3 ≥ Hb ≤ 3.0.

^**†**^Noise was calculated as the number of reads that were different from the called genotype divided by the total number of reads for the marker.

The low differentiation power of SNPs in mixtures is one of the biggest disadvantages compared to STRs^36^. Especially if the ratio of the major and minor contributors is highly skewed, it can be difficult to identify the presence of a mixed DNA sample^35,37^. Table 1 shows that there was a clear increase in the number of loci with a noise level higher than 3% for the 1:5, 1:6 and 1:18 mixtures. This indicates that many loci with high levels of noise is a clear indicator of a mixed sample, which was also observed previously for AmpliSeq assays^32^.

### Population genetics

Possible deviations from HWE were tested for the British, Syrian, and Middle Eastern individuals. After Bonferroni correction (p-value: 0.00031), all loci were in HWE. For the LD analysis, only individuals with full DNA profiles were used (Syria: N = 31, Middle East: N = 14, and Britain: N = 28). Rutgers Map Interpolator was used to infer distance in cM (compgen.rutgers.edu/map_interpolator.shtml). The LD test was performed for 13,203 combinations of loci. After Bonferroni correction (p-value = 3.79E−06), three pairs of loci displayed significant LD for the Syrian individuals: rs11746746 and rs4552703 (chromosome 5 and chromosome 6), rs2472304 and rs359955 (chromosome 15 and chromosome 1), and rs3099359 and rs424765 (chromosome 13 and chromosome 15). Two pairs of loci were in significant LD among the British individuals: rs1446585 and rs7570971 (both loci are on chromosome 2 separated by 0.28 cM), and rs1446585 and rs932206 (both loci are on chromosome 2 separated by 0.22 cM). Finally, two pairs of loci were in significant LD among the Middle Eastern individuals: rs10509722 and rs10994740 (both loci are on chromosome 10 separated by 40.07 cM), and rs310362 and rs39897 (chromosome 8 and chromosome 5).

**Figure 4:**
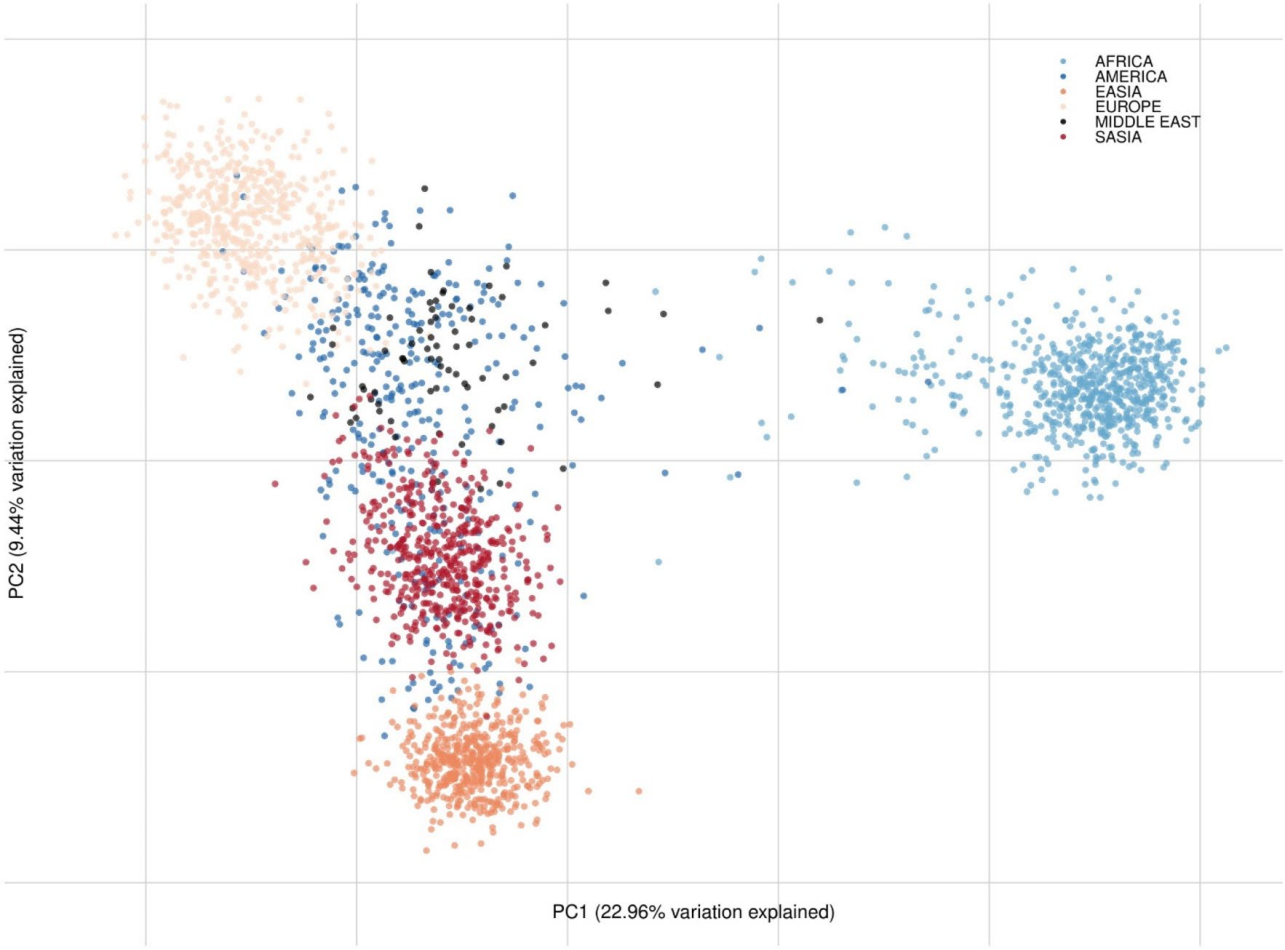
PCA plot of the studied populations and 1000 Genomes reference data. Meta-populations are listed in Table S2. ‘EASIA’ refers to East Asia and ‘SASIA’ refers to South-Central Asia.

The genetic relationships of the sequenced individuals and reference populations from 1000 Genomes (Supplementary Table S2) were visualized with PCA (Fig. 4). PC1 and PC2 described 24.23% and 8.94% of the variation, respectively. The reference African individuals all clustered together and were clearly separated from the rest of the populations. The loci in the QIAseq assay could also separate South Asian, European, and East Asian individuals. The Middle Eastern individuals typed here were located between the European, and South Asian individuals (Supplementary Fig. S5). The British individuals were clustered within the European reference populations as expected (Supplementary Fig. S4).

**Figure 5:**
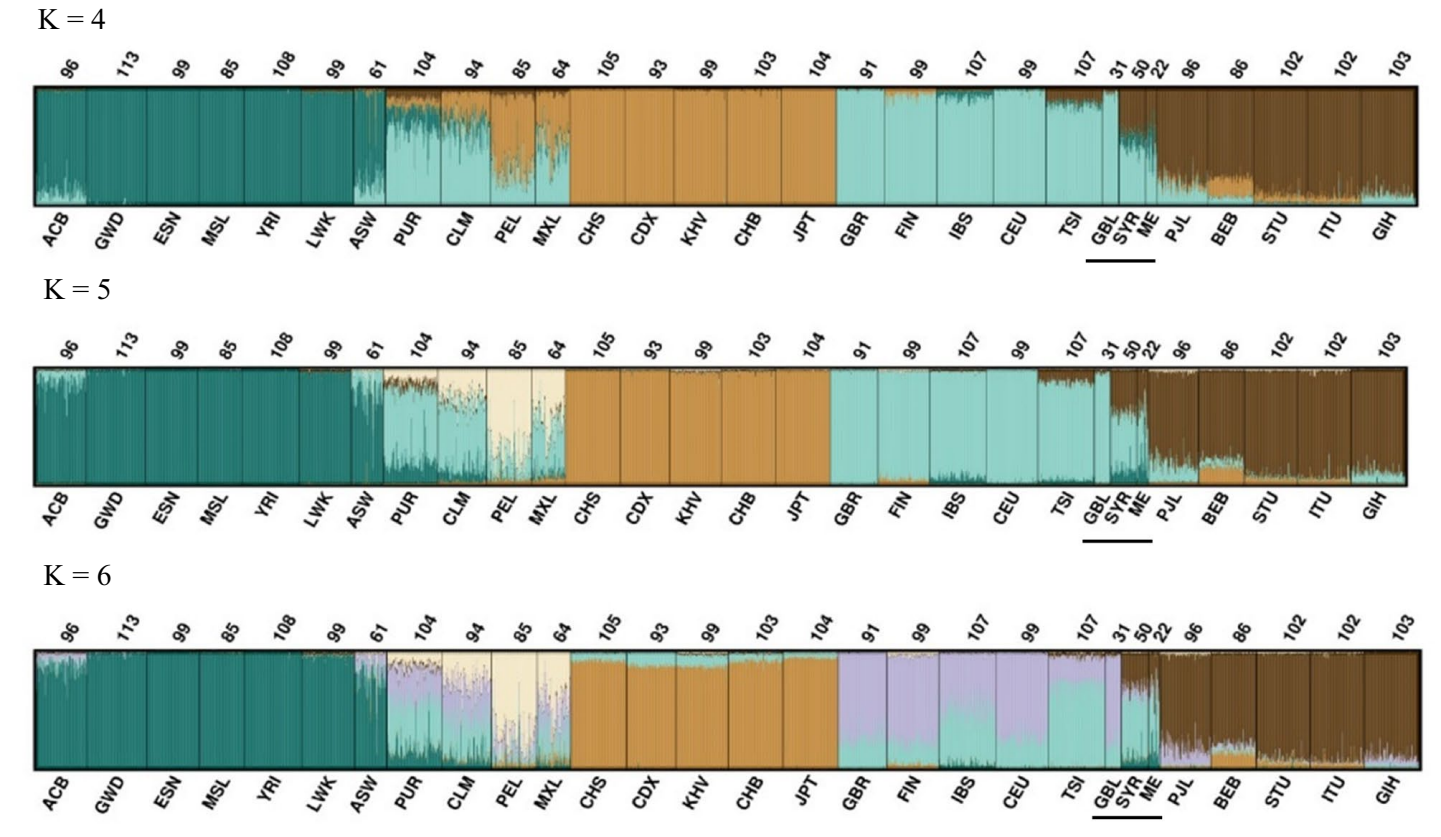
STRUCTURE plot with K = 4 to K = 6 using 163 SNPs. Numbers above each population refers to the sample size. Population abbreviations on the horizontal axis: Gambia, Africa (GWD), Esan, Nigeria (ESN), Mende, Sierra Leone (MSL), Yoruba, Ibadan, Nigeria (YRI), Luhya, Kenya (LWK), African American SW USA (ASW), Puerto Rican, Puerto Rico (PUR), Colombian in Medellin, (CLM), Peruvians, Lima, Peru (PEL), Mexican, Los Angeles (MXL), Southern Han Chinese, China (CHS), Chinese Dai, Xishuangbanna (CDX), Kinh, Vietnam (KHV), Han Chinese, Beijing, China (CHB), Japanese, Tokyo, Japan (JPT), British, England and Scotland (GBR), Finnish, Finland (FIN), Iberians, Spain (IBS), Utah residents, North and West European ancestry (CEU), Toscans, Italy (TSI), British individuals, Great Britain (GBL) from this study, Syrians, Syria (SYR) from this study, Middle East (ME) from this study, Punjabi, Lahore, Pakistan (PJL), Bengali, Bangladesh (BEB), Tamil, Sri Lanka, from United Kingdom (STU), Teluga, India, from United Kingdom (ITU), and Gujarati, India, Houston Texas (GIH). Populations with a black line below the name are the populations genotyped in this study.

The same test and reference data were analysed with STRUCTURE^38–41^ to investigate the ancestry of the individuals. Figure 5 shows the STRUCTURE results for K = 4 to K = 6 for 163 loci included in the QIAseq custom assay where K = 6 was the most likely number of clusters. At K = 5, clustering was observed between individuals from African, American, East Asian, European, and South-Central Asian populations. The Central and South American populations had high levels of European admixture. The British individuals shared cluster membership with the European reference population. Syrian and the Middle Eastern individuals primarily displayed European and South-Central Asian ancestry components. At K = 6, a new component was observed, that subdivided European individuals, reflecting northern (Great Britain and Finland) and southern European populations (Spanish and Italian).

The assay included a subset of loci that were previously tested (reference 15 and 24). The assay had fewer loci for European/Middle East differentiation compared to that of the EUROFORGEN NAME panel^15^, but on the other hand included more loci informative for differentiation of South-Central Asians. Differentiation between South-Central Asian populations and European populations was observed at K = 4. Separation of Middle Eastern populations from its surrounding regions of both Europe and South Asia was harder to obtain. Nevertheless, the markers in the QIAseq assay provided a better separation of the Middle Eastern cluster from the European and South-Central Asian clusters compared to those of global panels^4,19,42^. The STRUCTURE and PCA plots demonstrated the admixed nature of the Middle Eastern individuals analysed here. The Middle Eastern individuals shared the greatest affinity with the Southern than the Northern European components as seen by others^13,42,43^. Dividing the European population into Northern and Southern Europeans could potentially provide an increased differentiation between Northern Europe and the Middle East.

## Conclusions

The performance of the QIAseq assay showed consistent results when 10 to 40 ng DNA was used as specified in the protocol. Reliable results were also obtained with 2 ng DNA though with larger variation in the heterozygote balance. For DNA input below 2 ng, the assay displayed a high number of allele- and locus drop-outs. Here, the variation in heterozygote balance was high and around 10% of the genotypes displayed Hb outside the range of 0.3–3.0. Allele- and locus drop-outs were not consistent between replicates of the same concentration or between laboratories. Even loci that performed well experienced drop-outs, and it was, therefore, difficult to exclude poorly performing loci in the downstream analysis. The reduced sensitivity and large variation in read depth was likely due to the library preparation methods. The randomness in locus- and allele drop-outs indicated that the adapter ligation step was inefficient. The results showed that although the genotyping was reproducible for high input amounts of DNA, the QIAseq assay was not sufficiently sensitive for the low DNA concentrations often found in forensic genetics casework.

Samples with DNA from more than one contributor were easily detected by analysing the read counts. A high number of imbalanced heterozygous genotypes indicated a mixture with almost equal contributions from two people, whereas a high number of loci with noise above 3% indicated that the sample was a mixture with more skewed contributions from two people.

Except for three discordances of which two were due to locus drop-outs, there was full concordance between the SNP types of the Coriell samples typed with the QIAseq assay and the reference data.

Lastly, British and Middle Eastern individuals were typed for the loci in the QIAseq assay. The 163 markers included in the QIAseq assay provided a better separation of the Middle Eastern individuals than other reported panels. At K = 4, a South Asian cluster was easily identified. At K = 6, a Northern and Southern European gradient was identified. No distinct cluster for the Middle Eastern individuals was observed, and the greatest overlap was with the South European populations.

## Materials and methods

The work was performed as a collaboration between The University of Copenhagen (UCPH), University of Santiago de Compostela (USC), and King’s College, London (KCL).

### SNP markers

A custom-made panel consisting of 164 SNPs was used (Supplementary Table S1). The loci were selected for the differentiation of individuals from Europe, the Middle East, North Africa, and South Asia as previously described (reference 15 and 24). In addition to the EUROFORGEN NAME Panel^15^, the QIAseq assay also included loci for differentiating Europeans from South-Central Asians and North Africans.

### Samples, DNA extraction, and quantification

#### Sensitivity

To test the sensitivity of the QIAseq Targeted DNA Panel, a DNA dilution series was made from a Danish individual with known genotypes for the selected loci. The DNA was extracted from blood using the QIAamp DNA Blood Mini kit (Qiagen) following the manufacturer’s recommendations. The sample was quantified using the Quantifiler Trio DNA Quantification kit (Thermo Fisher Scientific) in quadruplicate and diluted to the following concentrations: 1 ng/µL, 0.2 ng/µL, 0.05 ng/µL, 0.025 ng/µL, and 0.0125 ng/µL. 10 µL of each sample was used for the library preparation. The dilution series was subsequently quantified using the Quantifiler Trio DNA Quantification kit (Thermo Fisher Scientific). The dilution series was sequenced once at KCL and in duplicate at UCPH.

#### Coriell reference and mixture samples

To evaluate the concordance between labs, four Coriell reference samples (1 ng/µL) with known genotypes and ancestry were analysed; two Europeans (CEU): NA06994 and NA07000, one African individual (YRI): NA18498, and one Asian individual (HCS): HG00403. Coriell samples are samples with known genotypes and ancestral background. The samples can be purchased from the Coriell Institute for Medical Research. USC was responsible for the purchase and distribution of the samples used in this study.

Mixture DNA was prepared from the Coriell samples NA06994 (CEU1) and NA07000 (CEU2) in the following ratios: 1:18, 1:6, 1:2, 3:2, and 5:1. DNA was quantified using the Qubit dsDNA High Sensitivity (HS) assay kit and the Qubit Fluorometer (Thermo Fisher Scientific) or the Quantifiler Trio DNA Quantification kit (Thermo Fisher Scientific). The Coriell samples and the DNA mixtures were sequenced once in both laboratories.

#### Population samples

Fifty unrelated Syrian individuals, 31 unrelated British individuals, and 22 unrelated Middle Eastern individuals were sequenced using the QIAseq custom panel. The Syrian samples were selected from the Biobank at the Department of Forensic Medicine, University of Copenhagen, Denmark. All samples were anonymised. The project was notified to the Ethics Committee for the Capital Region of Denmark (journal no. H-20006810). According to the Danish Act on Research Ethics Review of Health Research Projects, the work did not require approval by the Ethics Committee. DNA was extracted from buccal swabs on FTA cards (Whatman Inc., Clifton, NJ) with a BioRobot EZ1 workstation (Qiagen, Hilden, Germany) using the manufacturer’s protocol. The concentrations of the Syrian DNA extracts were measured using the Qubit dsDNA High Sensitivity (HS) assay kit and the Qubit Fluorometer (Thermo Fisher Scientific).

The British and Middle Eastern individuals were typed at KCL using the QIAseq custom panel. DNA was extracted using either Chelex 100 resin (Bio-Rad) or the QIAamp DNA Investigator Kit (Qiagen). DNA extracts were quantified using the Quantifiler Trio DNA Quantification kit (Thermo Fisher Scientific). Ethical approval for the study was granted by King’s College BDM Research Ethics Subcommittee (RESCM-18/19-2989) and all samples were collected with informed consent. The authors confirm that all methods were performed in accordance with the relevant guidelines and regulations.

A total of 5–10 ng DNA was used in the assay for all population samples.

### Library preparation

DNA libraries were built using the QIAseq Targeted DNA Panel kit (Qiagen) following the manufacturer’s protocol. The library kit and primer pool were kindly provided for testing by Qiagen. The DNA was enzymatically fragmented and end-repaired in the same reaction mix consisting of DNA (up to 16.75 µL), 2.5 µL Fragmentation Buffer 10x, 0.75 µL FERA solution, and a variable volume of nuclease-free water. The final reaction volume was 20 µL. Subsequently, 5 µL of Fragmentation Enzyme mix was added to each reaction. Samples were incubated for 1 min at 4 °C, 24 min at 32 °C, 30 min at 72 °C, and 4 °C on hold. Immediately after the fragmentation step, adapter ligation was performed by preparing a mix of 25 µL reaction mix from the previous step, 10 µL 5× Ligation Buffer, 7.2 µL Ligation solution, 2.8 µL IL-N7## adapter, and 5 µL DNA Ligase. Adapters were available with 12 different indices (IL-N701–N715), which combined with the 8 different Index primers (IL-S502–S511) gave a multiplex capacity of 96 samples. The reactions were incubated for 15 min at room temperature. The ligated adapters contain the UMIs and the sample index. After the sequencing run of the dilution series, a large number of adapter dimers were observed, when the DNA input was lower than specified in the protocol. To avoid adapter dimers and to maintain a balance between adapters and sample DNA, the amount of adapters had to be reduced when working with lower input than that specified in the protocol. When adjusting the amount of adapter for the samples with a lower DNA amount than specified in the protocol, the adapter dimer formation decreased markedly (Supplementary Fig. S6). To accommodate for this, adapters were diluted 10 times with nuclease-free water for the 2 ng, 0.5 ng, 0.25 ng, and 0.125 ng input DNA in the sensitivity study (Supplementary Fig. S6). The adapters were diluted twice for the Syrian individuals.

Following the adapter ligation step, a double clean-up was performed using QIAseq beads. Target enrichment was performed by preparing: 9.4 µL adapter-ligated DNA from the previous step, 4 µL 5 × TEPCR buffer, 5 µL QIAseq Targeted DNA Panel, 0.8 µL IL-Forward primer, and 0.8 µL HotStarTaq DNA Polymerase (Qiagen). The following cycling conditions were used for the first PCR: initial denaturation for 13 min at 95 °C and 2 min at 98 °C, 8 cycles of 15 s at 98 °C and 10 min at 68 °C, 1 cycle of 5 min at 72 °C, 1 hold cycle for 5 min at 4 °C, and hold at 4 °C. Following the target enrichment, a clean-up was performed using QIAseq beads. The second PCR, a universal PCR, was performed by adding a mix of 4 µL 5× UPCR buffer, 1 µL HotStarTaq DNA Polymerase, and 1.6 µL nuclease-free water to each of the target-enriched libraries. A total of 20 µL PCR products were transferred to the QIAseq 96-index I set A plate, which contained the pre-dispensed sample index primer (IL-S502–S511) and the universal primer. The following cycling conditions were used for the second PCR: initial denaturation 13 min at 95 °C and 2 min at 98 °C, 22 cycles for 15 s at 98 °C and 2 min at 60 °C, 1 cycle for 5 min at 72 °C, 1 hold for 5 min at 4 °C, and hold at 4 °C. Following the Universal PCR, a clean-up was performed using QIAseq beads.

### Sequencing

At UCPH, quantification of the purified libraries was performed using the Qubit dsDNA High Sensitivity (HS) assay kit and the Qubit 2.0 Fluorometer (Thermo Fisher Scientific). At KCL, libraries were quantified using the Kapa Library Quantification Kit (Roche Sequencing). After quantification, the libraries were normalized, pooled, and diluted to 2 nM. The pool was further diluted to either 10, 12, or 13 pM. Sequencing was performed with the Illumina MiSeq according to manufacturer’s recommendations using paired-end sequencing (2 × 150 bp) with the MiSeq v.2 reagent kit and a custom primer (Custom Read primer 1) provided with the QIAseq library kit.

### Data analysis

Post-sequencing analysis of the FASTQ files was performed using the Genomics Workbench v. 12.0.3 (Qiagen) with the Biomedical Genomics Analysis Plugin v. 1.2.1, corresponding target and hotspot BED-files for the hg19 reference genome. Briefly, UMIs were trimmed, and reads were annotated with the corresponding UMI. UMI annotated reads were mapped to hg19, and a single consensus read, a UMI read, was created from aligned reads that had the same UMI. A minimum of 10 UMI reads per locus was used as threshold. Subsequently, ligation artefacts (when two DNA sequences were ligated) were removed. Potential structural variants and indels were detected and annotated. Using the annotation of structural variants and indels, a local realignment was performed to improve the alignment of the reads. Next, known variants were identified, and a .csv file with the called variants was inspected. For the KCL data, the UMIs were removed during the ‘Adapter removal’ analysis step and it was therefore not possible to create consensus UMI reads for this dataset. The overall read depth was, therefore, higher for the KCL dataset. The workflow for the KCL data was identical to the UCPH workflow with the exception that all UMI processing steps were removed. The UMIs are used to collapse all reads with the same UMI into consensus UMI reads. The number of UMIs per locus for the sensitivity study can be found in Supplementary Table S6. Even though the addition of UMIs to DNA fragments is part of the library preparation in the QIAseq assay, they are not relevant to the analyses performed in this study as the genotypes were known in advance.

Genotype calling parameters were: minimum read depth = 20 reads, acceptable homozygote genotype calls with less than 10% noise, and acceptable heterozygote balance (Hb) was 0.3 to 3.0. Hb was calculated as the number of reads for one nucleotide divided by the number of reads for the other nucleotide in the called genotype in the order A, C, G, and T. All genotypes that did not meet these requirements were changed to NN. A maximum noise level of 3% was used in the mixture analysis. The noise was estimated as the number of reads that were different from the called genotype divided by the total number of reads for the marker in question.

### Population genetics analysis

Deviations from Hardy–Weinberg expectations were estimated using Arlequin v.3.5.2.2^44^ and 1,000,000 Markov chain steps. Arlequin was also used to test for pairwise linkage disequilibrium (LD) using an exact test. Alpha was adjusted according to the Bonferroni correction^45^.

Principal component analysis (PCA) was performed with a custom script written in R v. 3.5.0 and the ‘*adegenet’* and the ‘*ade4*’ R packages^46,47^. STRUCTURE^38–41^ analysis was carried out to assess how good the selected markers were at inferring ancestry components of each sample. ‘Admixture’ and ‘correlated allele frequency’ models were employed with the labelling of reference populations by the use of POPFLAG = 1. The STRUCTURE analysis was performed using 100,000 steps of burn-in followed by 100,000 MCMC steps. Three to seven clusters (K) were considered with five independent runs per K. Structure Harvester was used to find the most likely number of K^48^. The software CLUMPP v.1.1.2.^49^ was used to combine the information from several independent STRUCTURE runs for the same number of clusters. The output matrix is a mean of the permuted matrices across replicates. This output from CLUMPP was visualised by Distruct v.1.1^50^. Reference data were obtained from the 1000 Genomes database^51^ (Supplementary Table S2). Marker rs10907192 was not considered in the analysis since no reference data were available.

## Supplementary Information

Supplementary Information.
