# Supplementary material for: Evaluation of a custom QIAseq targeted DNA panel with 164 ancestry informative markers sequenced with the Illumina MiSeq
Source: Sci Rep. 2021 Oct 26;11:21040. doi: 10.1038/s41598-021-99933-2 (PMC8548529; doi:10.1038/s41598-021-99933-2)
Supplement: Supplementary file 1 — Supplementary Information. [file 41598_2021_99933_MOESM1_ESM.docx]

**Supplementary materials for:**

*Evaluation of a custom QIAseq^TM^ Targeted DNA Panel with 164 ancestry informative markers sequenced with the Illumina MiSeq.*

D.M. Truelsen^a*^, A.Freire-Aradas^b^, M. Nazari^c^, A. Aliferi^c^, D. Ballard^c^, C. Phillips^b^, N. Morling^a,d^, V. Pereira^a^, C. Børsting^a^

^a^ Section of Forensic Genetics, Department of Forensic Medicine, Faculty of Health and Medical Sciences, University of Copenhagen, 2100 Copenhagen, Denmark

^b^ Forensic Genetics Unit, Institute of Forensic Sciences, University of Santiago de Compostela, Spain

^c^ Faculty of Life Sciences and Medicine, King’s College, London, UK

^d^ Department of Mathematical Sciences, Aalborg University, DK-9220 Aalborg East, Denmark

* E-mail corresponding author: forensic.genetics@sund.ku.dk

**Supplementary materials**

*Evaluation of a custom QIAseq^TM^ Targeted DNA Panel with 164 ancestry informative markers sequenced with the Illumina MiSeq.*

**Table S1**: List of loci included in the QIAseq assay.

| **SNP** | **Chromosome** | **Position (hg19)** |
| --- | --- | --- |
| rs10907192 | 1 | 1793111 |
| rs359955 | 1 | 33134774 |
| rs7354930 | 1 | 66986377 |
| rs2205848 | 1 | 169676293 |
| rs6701640 | 1 | 170696474 |
| rs2227203 | 1 | 172879023 |
| rs17692475 | 1 | 193808319 |
| rs1470637 | 1 | 205033283 |
| rs6673820 | 1 | 205910604 |
| rs12070887 | 1 | 230012316 |
| rs608825 | 1 | 236568770 |
| rs947178 | 1 | 247524439 |
| rs10736372 | 1 | 247977420 |
| rs7594173 | 2 | 32900330 |
| rs6741107 | 2 | 38652572 |
| rs10179227 | 2 | 69330445 |
| rs1519654 | 2 | 101096960 |
| rs6759439 | 2 | 107801393 |
| rs621341 | 2 | 135295180 |
| rs7570971 | 2 | 135837906 |
| rs1446585 | 2 | 136407479 |
| rs932206 | 2 | 136825272 |
| rs4668060 | 2 | 169166305 |
| rs6805861 | 3 | 465476 |
| rs17349171 | 3 | 65226990 |
| rs984038 | 3 | 65762252 |
| rs9817359 | 3 | 76473163 |
| rs1002765 | 3 | 87277062 |
| rs13064646 | 3 | 144516564 |
| rs9877982 | 3 | 181602745 |
| rs1994855 | 3 | 192067566 |
| rs2291652 | 3 | 195477791 |
| rs718501 | 3 | 197083723 |
| rs4833103 | 4 | 38815502 |

Table S1 continued

| **SNP** | **Chromosome** | **Position (hg19)** |
| --- | --- | --- |
| rs4834738 | 4 | 119980752 |
| rs12508176 | 4 | 123997900 |
| rs4975270 | 4 | 129757215 |
| rs1757928 | 4 | 130022161 |
| rs3796564 | 4 | 156688928 |
| rs4956993 | 5 | 184702 |
| rs28777 | 5 | 33958959 |
| rs2172771 | 5 | 56718875 |
| rs16891097 | 5 | 62091796 |
| rs3097146 | 5 | 87498849 |
| rs636137 | 5 | 106806224 |
| rs6894681 | 5 | 127218995 |
| rs11746746 | 5 | 127682184 |
| rs39897 | 5 | 131436896 |
| rs4308478 | 5 | 136334314 |
| rs262037 | 5 | 177990886 |
| rs1023056 | 6 | 21988752 |
| rs9467370 | 6 | 24968682 |
| rs12203115 | 6 | 36490733 |
| rs11963228 | 6 | 73733188 |
| rs4552703 | 6 | 91486381 |
| rs9487258 | 6 | 110263644 |
| rs17086288 | 6 | 124210612 |
| rs9493012 | 6 | 131843741 |
| rs6901197 | 6 | 145097563 |
| rs6919370 | 6 | 151048139 |
| rs9479657 | 6 | 153928396 |
| rs1853025 | 6 | 161144729 |
| rs1357681 | 7 | 10511733 |
| rs3852253 | 7 | 18866190 |
| rs10242595 | 7 | 22774231 |
| rs2255365 | 7 | 24561661 |
| rs848461 | 7 | 77582265 |
| rs2966487 | 7 | 112334945 |
| rs10278248 | 7 | 112802475 |
| rs7803075 | 7 | 130742066 |
| rs1495085 | 8 | 15298515 |
| rs11779571 | 8 | 18544452 |
| rs4737753 | 8 | 54701811 |

Table S1 continued

| **SNP** | **Chromosome** | **Position (hg19)** |
| --- | --- | --- |
| rs310362 | 8 | 59925618 |
| rs7816786 | 8 | 101349662 |
| rs964307 | 8 | 110413762 |
| rs7814782 | 8 | 121629520 |
| rs2196051 | 8 | 122124302 |
| rs6989963 | 8 | 128643231 |
| rs7025610 | 9 | 753093 |
| rs10960718 | 9 | 12606092 |
| rs10810656 | 9 | 16877758 |
| rs11144480 | 9 | 78158285 |
| rs1110403 | 9 | 110383583 |
| rs7872110 | 9 | 137098042 |
| rs487750 | 9 | 138603740 |
| rs2031581 | 10 | 4069527 |
| rs1974068 | 10 | 14815820 |
| rs12414926 | 10 | 15674005 |
| rs1733743 | 10 | 56847522 |
| rs10994740 | 10 | 63127639 |
| rs875001 | 10 | 72984697 |
| rs7922818 | 10 | 85715936 |
| rs6586103 | 10 | 89584002 |
| rs10509722 | 10 | 100278017 |
| rs603424 | 10 | 102075479 |
| rs7075775 | 10 | 122099219 |
| rs7921384 | 10 | 127414585 |
| rs4910031 | 11 | 11922325 |
| rs7102164 | 11 | 17572284 |
| rs1451393 | 11 | 83516681 |
| rs1785864 | 11 | 104955489 |
| rs7947221 | 11 | 109241241 |
| rs1941411 | 11 | 120130512 |
| rs7975017 | 12 | 26428793 |
| rs1133028 | 12 | 28603112 |
| rs6582668 | 12 | 38766604 |
| rs10506882 | 12 | 83205152 |
| rs10507055 | 12 | 95777318 |
| rs1544656 | 12 | 113008333 |
| rs9315120 | 13 | 31823589 |
| rs2182216 | 13 | 46337299 |

Table S1 continued

| **SNP** | **Chromosome** | **Position (hg19)** |
| --- | --- | --- |
| rs7981142 | 13 | 51589685 |
| rs3099359 | 13 | 96485417 |
| rs6492155 | 13 | 109570601 |
| rs9522149 | 13 | 111827167 |
| rs11840507 | 13 | 112737954 |
| rs7148809 | 14 | 65680885 |
| rs2295111 | 14 | 68236522 |
| rs12880237 | 14 | 68621818 |
| rs10131666 | 14 | 74212431 |
| rs6574774 | 14 | 84616115 |
| rs927140 | 14 | 94623337 |
| rs11636232 | 15 | 28386626 |
| rs1024124 | 15 | 33617064 |
| rs3092981 | 15 | 40987725 |
| rs2472304 | 15 | 75044238 |
| rs6496996 | 15 | 93402496 |
| rs8027443 | 15 | 100285705 |
| rs6500567 | 16 | 4083105 |
| rs896401 | 16 | 8454655 |
| rs166054 | 16 | 11285202 |
| rs17625895 | 16 | 25775102 |
| rs7198400 | 16 | 78431277 |
| rs4782726 | 16 | 82701333 |
| rs9899480 | 17 | 36185665 |
| rs4465645 | 17 | 50832843 |
| rs1564821 | 17 | 53248927 |
| rs1549519 | 17 | 53757787 |
| rs8064588 | 17 | 58361461 |
| rs9907597 | 17 | 59435497 |
| rs11652975 | 17 | 72288399 |
| rs4796918 | 18 | 11286964 |
| rs2337024 | 18 | 20875793 |
| rs10503052 | 18 | 58899633 |
| rs2156208 | 18 | 60131306 |
| rs17080463 | 18 | 66810095 |
| rs3862700 | 18 | 67862224 |
| rs1363345 | 19 | 13574131 |
| rs11668441 | 19 | 31803670 |
| rs10401746 | 19 | 39320960 |

Table S1 continued

| **SNP** | **Chromosome** | **Position (hg19)** |
| --- | --- | --- |
| rs7252391 | 19 | 44142771 |
| rs307896 | 19 | 47661493 |
| rs10419435 | 19 | 56312145 |
| rs6081765 | 20 | 2035775 |
| rs6071173 | 20 | 37336474 |
| rs1296032 | 20 | 51829213 |
| rs3852924 | 20 | 54059074 |
| rs234623 | 20 | 57488964 |
| rs6026972 | 20 | 58147511 |
| rs2822793 | 21 | 15955911 |
| rs2835133 | 21 | 37133457 |
| rs424765 | 22 | 18486017 |
| rs564666 | 22 | 27678463 |

Table S2: Reference populations from 1000 Genomes project and populations typed with the QIAseq assay.

| **Population** | **Abbreviation** | **Meta-population** | **Number of individuals** |
| --- | --- | --- | --- |
| African Caribbean in Barbados | ACB | Africa | 96 |
| Gambian in Western Division | GWD |  | 113 |
| Esan in Nigeria | ESN |  | 99 |
| Mende in Sierra Leone | MSL |  | 85 |
| Yoruba in Ibadan | YRI |  | 108 |
| Luhya in Webuye | LWK |  | 99 |
| African Ancestry in Southwest US | ASW |  | 61 |
| Puerto Rican in Puerto Rico | PUR | America | 104 |
| Colombian in Medellin | CLM |  | 94 |
| Peruvian in Lima | PEL |  | 85 |
| Mexican Ancestry in Los Angeles | MXL |  | 64 |
| Southern Han Chinese | CHS | East Asia | 105 |
| Chinese Dai in Xishuangbanna | CDX |  | 93 |
| Kinh in Ho Chi Minh City | KHV |  | 99 |
| Han Chinese in Bejing | CHB |  | 103 |
| Japanese in Tokyo | JPT |  | 104 |
| British in England and Scotland | GBR | Europe | 91 |
| Finnish in Finland | FIN |  | 99 |
| Iberian populations in Spain | IBS |  | 107 |
| Utah residents with Northern and Western European ancestry | CEU |  | 99 |
| Toscani in Italy | TSI |  | 107 |
| British individuals^a^ | GBL |  | 31 |
| Syrians^a^ | SYR | Middle East | 50 |
| Middle Easterners^a^ | ME |  | 22 |
| Punjabi in Lahore | PJL | South-Central Asia | 96 |
| Bengali in Bangladesh | BEB |  | 86 |
| Sri Lankan Tamil in the UK | STU |  | 102 |
| Indian Telugu in the UK | ITU |  | 102 |
| Gujarati Indian in Houston | GIH |  | 103 |

*^a^*Populations typed in this study.

Table S3: Number of times a locus displayed locus drop-out in the sensitivity study in % for all dilutions (15 dilutions in total), for dilutions typed at UCPH (2 replicates of each dilution = 10 dilutions), and for dilutions typed at KCL (5 dilutions).

| **Locus** | **Total number of locus drop-outs in %** | **Drop-outs for UCPH in % ^a^** | **Drop-outs for KCL in % ^b^** |
| --- | --- | --- | --- |
| rs718501 | 60.0 | 60.0 | 60.0 |
| rs1470637 | 46.7 | 50.0 | 40.0 |
| rs1733743 | 46.7 | 60.0 | 20.0 |
| rs896401 | 46.7 | 40.0 | 60.0 |
| rs12880237 | 40.0 | 50.0 | 20.0 |
| rs3796564 | 40.0 | 50.0 | 20.0 |
| rs6901197 | 40.0 | 40.0 | 40.0 |
| rs964307 | 40.0 | 40.0 | 40.0 |
| rs17625895 | 33.3 | 30.0 | 40.0 |
| rs2196051 | 33.3 | 50.0 | 0.0 |
| rs2205848 | 33.3 | 40.0 | 20.0 |
| rs2966487 | 33.3 | 40.0 | 20.0 |
| rs4552703 | 33.3 | 40.0 | 20.0 |
| rs6026972 | 33.3 | 40.0 | 20.0 |
| rs6894681 | 33.3 | 30.0 | 40.0 |
| rs7872110 | 33.3 | 30.0 | 40.0 |
| rs7975017 | 33.3 | 40.0 | 20.0 |
| rs984038 | 33.3 | 50.0 | 0.0 |
| rs1002765 | 26.7 | 20.0 | 40.0 |
| rs10503052 | 26.7 | 20.0 | 40.0 |
| rs10507055 | 26.7 | 20.0 | 40.0 |
| rs11746746 | 26.7 | 20.0 | 40.0 |
| rs11840507 | 26.7 | 30.0 | 20.0 |
| rs11963228 | 26.7 | 30.0 | 20.0 |
| rs12203115 | 26.7 | 30.0 | 20.0 |
| rs13064646 | 26.7 | 40.0 | 0.0 |
| rs17086288 | 26.7 | 30.0 | 20.0 |
| rs17692475 | 26.7 | 20.0 | 40.0 |
| rs1785864 | 26.7 | 30.0 | 20.0 |
| rs1974068 | 26.7 | 30.0 | 20.0 |
| rs2172771 | 26.7 | 30.0 | 20.0 |
| rs2227203 | 26.7 | 30.0 | 20.0 |
| rs2822793 | 26.7 | 30.0 | 20.0 |
| rs2835133 | 26.7 | 30.0 | 20.0 |
| rs4796918 | 26.7 | 30.0 | 20.0 |
| rs4834738 | 26.7 | 20.0 | 40.0 |
| rs6071173 | 26.7 | 40.0 | 0.0 |
| rs6500567 | 26.7 | 30.0 | 20.0 |

Table S3 continued

| **Locus** | **Total number of locus drop-outs in %** | **Drop-outs for UCPH in % ^a^** | **Drop-outs for KCL in % ^b^** |
| --- | --- | --- | --- |
| rs6574774 | 26.7 | 30.0 | 20.0 |
| rs6582668 | 26.7 | 20.0 | 40.0 |
| rs6919370 | 26.7 | 30.0 | 20.0 |
| rs6989963 | 26.7 | 30.0 | 20.0 |
| rs7148809 | 26.7 | 30.0 | 20.0 |
| rs7814782 | 26.7 | 40.0 | 0.0 |
| rs8027443 | 26.7 | 40.0 | 0.0 |
| rs9315120 | 26.7 | 30.0 | 20.0 |
| rs9522149 | 26.7 | 30.0 | 20.0 |
| rs10179227 | 20.0 | 20.0 | 20.0 |
| rs1023056 | 20.0 | 20.0 | 20.0 |
| rs1024124 | 20.0 | 30.0 | 0.0 |
| rs10278248 | 20.0 | 20.0 | 20.0 |
| rs10401746 | 20.0 | 30.0 | 0.0 |
| rs10419435 | 20.0 | 20.0 | 20.0 |
| rs10506882 | 20.0 | 20.0 | 20.0 |
| rs10509722 | 20.0 | 20.0 | 20.0 |
| rs10736372 | 20.0 | 30.0 | 0.0 |
| rs10810656 | 20.0 | 20.0 | 20.0 |
| rs10907192 | 20.0 | 30.0 | 0.0 |
| rs11652975 | 20.0 | 20.0 | 20.0 |
| rs11779571 | 20.0 | 30.0 | 0.0 |
| rs12508176 | 20.0 | 30.0 | 0.0 |
| rs1363345 | 20.0 | 30.0 | 0.0 |
| rs1519654 | 20.0 | 20.0 | 20.0 |
| rs1549519 | 20.0 | 20.0 | 20.0 |
| rs16891097 | 20.0 | 20.0 | 20.0 |
| rs17080463 | 20.0 | 10.0 | 40.0 |
| rs1941411 | 20.0 | 30.0 | 0.0 |
| rs1994855 | 20.0 | 30.0 | 0.0 |
| rs2255365 | 20.0 | 20.0 | 20.0 |
| rs2295111 | 20.0 | 20.0 | 20.0 |
| rs262037 | 20.0 | 30.0 | 0.0 |
| rs3092981 | 20.0 | 20.0 | 20.0 |
| rs3099359 | 20.0 | 30.0 | 0.0 |
| rs359955 | 20.0 | 20.0 | 20.0 |
| rs3852253 | 20.0 | 20.0 | 20.0 |
| rs3862700 | 20.0 | 20.0 | 20.0 |

Table S3 continued

| **Locus** | **Total number of locus drop-outs in %** | **Drop-outs for UCPH in % ^a^** | **Drop-outs for KCL in % ^b^** |
| --- | --- | --- | --- |
| rs424765 | 20.0 | 20.0 | 20.0 |
| rs4465645 | 20.0 | 30.0 | 0.0 |
| rs4782726 | 20.0 | 30.0 | 0.0 |
| rs4833103 | 20.0 | 30.0 | 0.0 |
| rs4956993 | 20.0 | 30.0 | 0.0 |
| rs4975270 | 20.0 | 20.0 | 20.0 |
| rs564666 | 20.0 | 20.0 | 20.0 |
| rs603424 | 20.0 | 20.0 | 20.0 |
| rs621341 | 20.0 | 20.0 | 20.0 |
| rs636137 | 20.0 | 20.0 | 20.0 |
| rs6492155 | 20.0 | 30.0 | 0.0 |
| rs6759439 | 20.0 | 30.0 | 0.0 |
| rs7075775 | 20.0 | 30.0 | 0.0 |
| rs7102164 | 20.0 | 20.0 | 20.0 |
| rs7198400 | 20.0 | 20.0 | 20.0 |
| rs7252391 | 20.0 | 20.0 | 20.0 |
| rs7354930 | 20.0 | 30.0 | 0.0 |
| rs7570971 | 20.0 | 30.0 | 0.0 |
| rs7922818 | 20.0 | 30.0 | 0.0 |
| rs927140 | 20.0 | 30.0 | 0.0 |
| rs932206 | 20.0 | 20.0 | 20.0 |
| rs9487258 | 20.0 | 30.0 | 0.0 |
| rs9493012 | 20.0 | 30.0 | 0.0 |
| rs9817359 | 20.0 | 20.0 | 20.0 |
| rs9877982 | 20.0 | 30.0 | 0.0 |
| rs10960718 | 13.3 | 20.0 | 0.0 |
| rs11144480 | 13.3 | 20.0 | 0.0 |
| rs1133028 | 13.3 | 20.0 | 0.0 |
| rs11636232 | 13.3 | 10.0 | 20.0 |
| rs11668441 | 13.3 | 10.0 | 20.0 |
| rs12414926 | 13.3 | 20.0 | 0.0 |
| rs1296032 | 13.3 | 20.0 | 0.0 |
| rs1446585 | 13.3 | 20.0 | 0.0 |
| rs1451393 | 13.3 | 20.0 | 0.0 |
| rs1544656 | 13.3 | 20.0 | 0.0 |
| rs166054 | 13.3 | 20.0 | 0.0 |
| rs1757928 | 13.3 | 10.0 | 20.0 |
| rs2156208 | 13.3 | 10.0 | 20.0 |

Table S3 continued

| **Locus** | **Total number of locus drop-outs in %** | **Drop-outs for UCPH in % ^a^** | **Drop-outs for KCL in % ^b^** |
| --- | --- | --- | --- |
| rs2182216 | 13.3 | 20.0 | 0.0 |
| rs2337024 | 13.3 | 20.0 | 0.0 |
| rs234623 | 13.3 | 20.0 | 0.0 |
| rs28777 | 13.3 | 20.0 | 0.0 |
| rs307896 | 13.3 | 10.0 | 20.0 |
| rs3097146 | 13.3 | 20.0 | 0.0 |
| rs310362 | 13.3 | 10.0 | 20.0 |
| rs3852924 | 13.3 | 10.0 | 20.0 |
| rs39897 | 13.3 | 20.0 | 0.0 |
| rs4308478 | 13.3 | 20.0 | 0.0 |
| rs4668060 | 13.3 | 10.0 | 20.0 |
| rs4737753 | 13.3 | 20.0 | 0.0 |
| rs4910031 | 13.3 | 20.0 | 0.0 |
| rs6496996 | 13.3 | 20.0 | 0.0 |
| rs6701640 | 13.3 | 10.0 | 20.0 |
| rs6805861 | 13.3 | 10.0 | 20.0 |
| rs7025610 | 13.3 | 20.0 | 0.0 |
| rs7594173 | 13.3 | 20.0 | 0.0 |
| rs7803075 | 13.3 | 20.0 | 0.0 |
| rs7921384 | 13.3 | 20.0 | 0.0 |
| rs7947221 | 13.3 | 10.0 | 20.0 |
| rs7981142 | 13.3 | 20.0 | 0.0 |
| rs8064588 | 13.3 | 20.0 | 0.0 |
| rs848461 | 13.3 | 20.0 | 0.0 |
| rs875001 | 13.3 | 20.0 | 0.0 |
| rs9467370 | 13.3 | 20.0 | 0.0 |
| rs947178 | 13.3 | 20.0 | 0.0 |
| rs9899480 | 13.3 | 20.0 | 0.0 |
| rs9907597 | 13.3 | 10.0 | 20.0 |
| rs10131666 | 6.7 | 10.0 | 0.0 |
| rs10242595 | 6.7 | 10.0 | 0.0 |
| rs10994740 | 6.7 | 10.0 | 0.0 |
| rs1110403 | 6.7 | 10.0 | 0.0 |
| rs1357681 | 6.7 | 10.0 | 0.0 |
| rs1495085 | 6.7 | 10.0 | 0.0 |
| rs1564821 | 6.7 | 10.0 | 0.0 |
| rs17349171 | 6.7 | 10.0 | 0.0 |
| rs1853025 | 6.7 | 10.0 | 0.0 |

Table S3 continued

| **Locus** | **Total number of locus drop-outs in %** | **Drop-outs for UCPH in % ^a^** | **Drop-outs for KCL in % ^b^** |
| --- | --- | --- | --- |
| rs2031581 | 6.7 | 10.0 | 0.0 |
| rs2291652 | 6.7 | 10.0 | 0.0 |
| rs2472304 | 6.7 | 10.0 | 0.0 |
| rs487750 | 6.7 | 10.0 | 0.0 |
| rs6081765 | 6.7 | 10.0 | 0.0 |
| rs608825 | 6.7 | 10.0 | 0.0 |
| rs6741107 | 6.7 | 10.0 | 0.0 |
| rs7816786 | 6.7 | 10.0 | 0.0 |
| rs9479657 | 6.7 | 10.0 | 0.0 |

^a^ For ten samples (each dilution run in duplicates)

^b^ For five samples (each dilution run once)


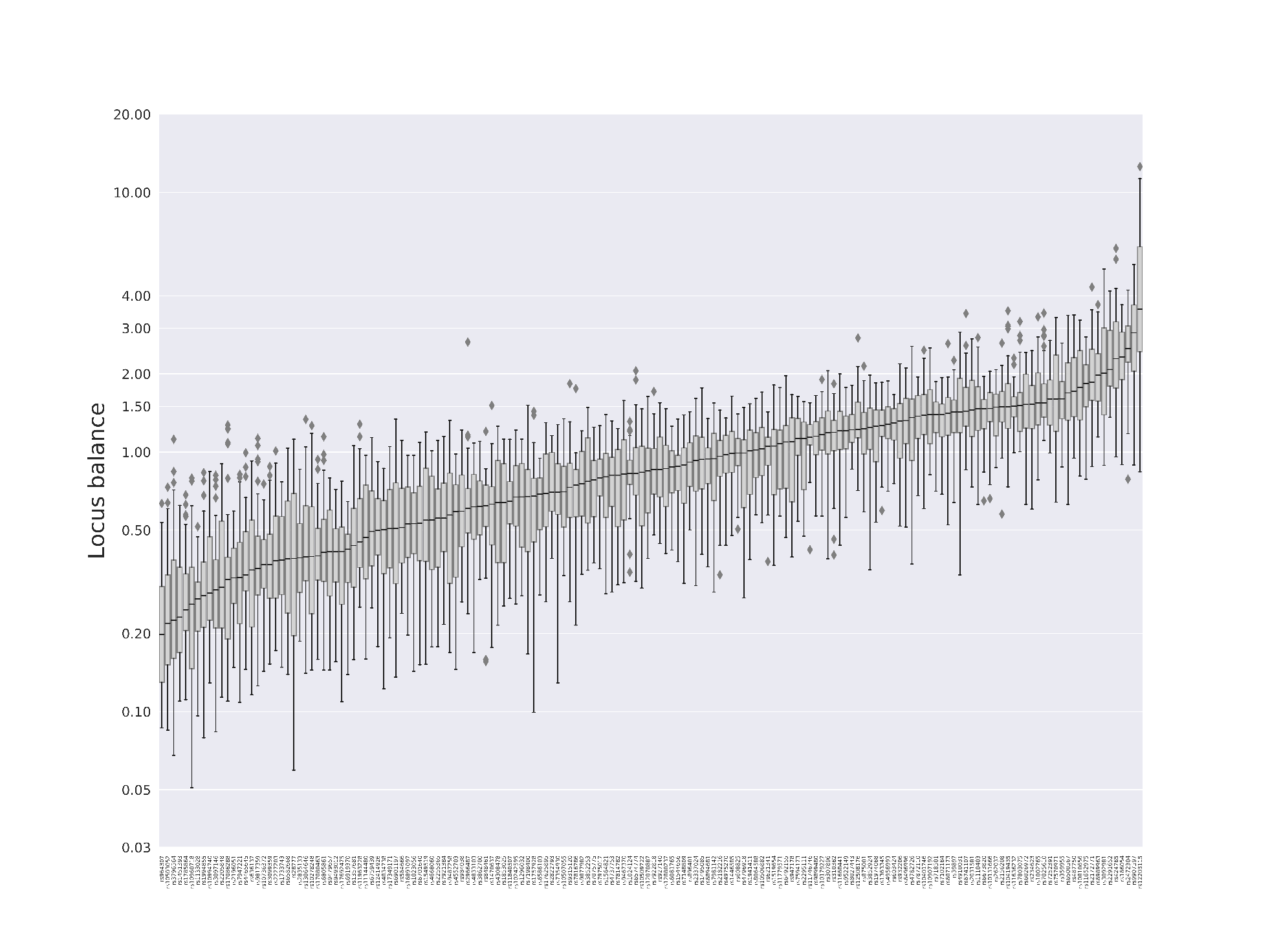


Fig. S1: Locus balance for the 50 Syrians sequenced for the 164 SNPs included in the QIAseq assay. Locus balance was calculated as the number of reads for one locus divided by the average number of reads among all loci. Data were not filtered to allow the direct assessment of the locus balance.


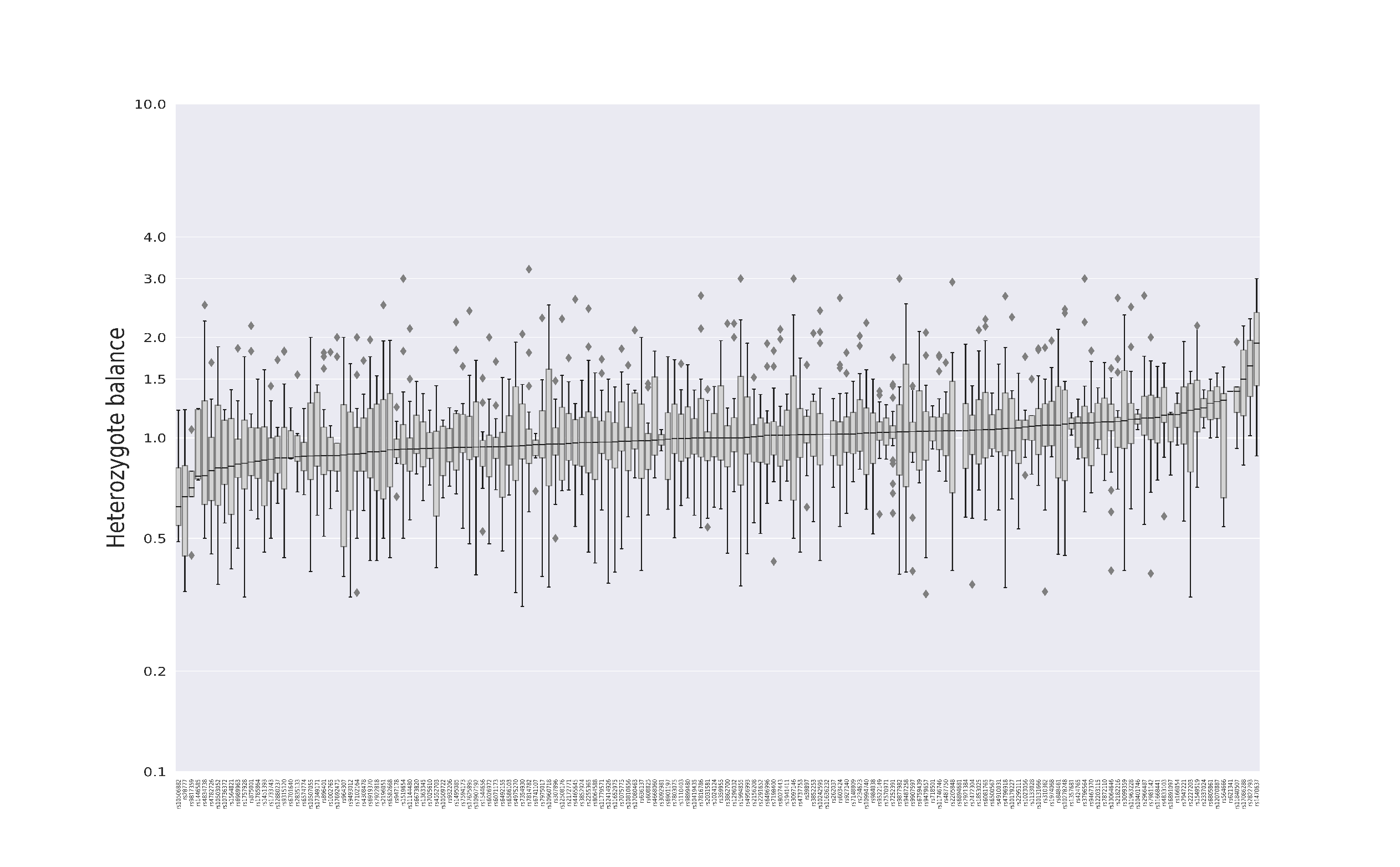


Fig. S2: Heterozygote balance (Hb) for the 50 Syrians sequenced for the 164 SNPs included in the QIAseq assay. Heterozygote balance was estimated as the number of reads for one nucleotide divided by the number of reads for the other nucleotide in the following order: A, C, G, and T. Data were not filtered to allow the direct assessment of the heterozygote balance.


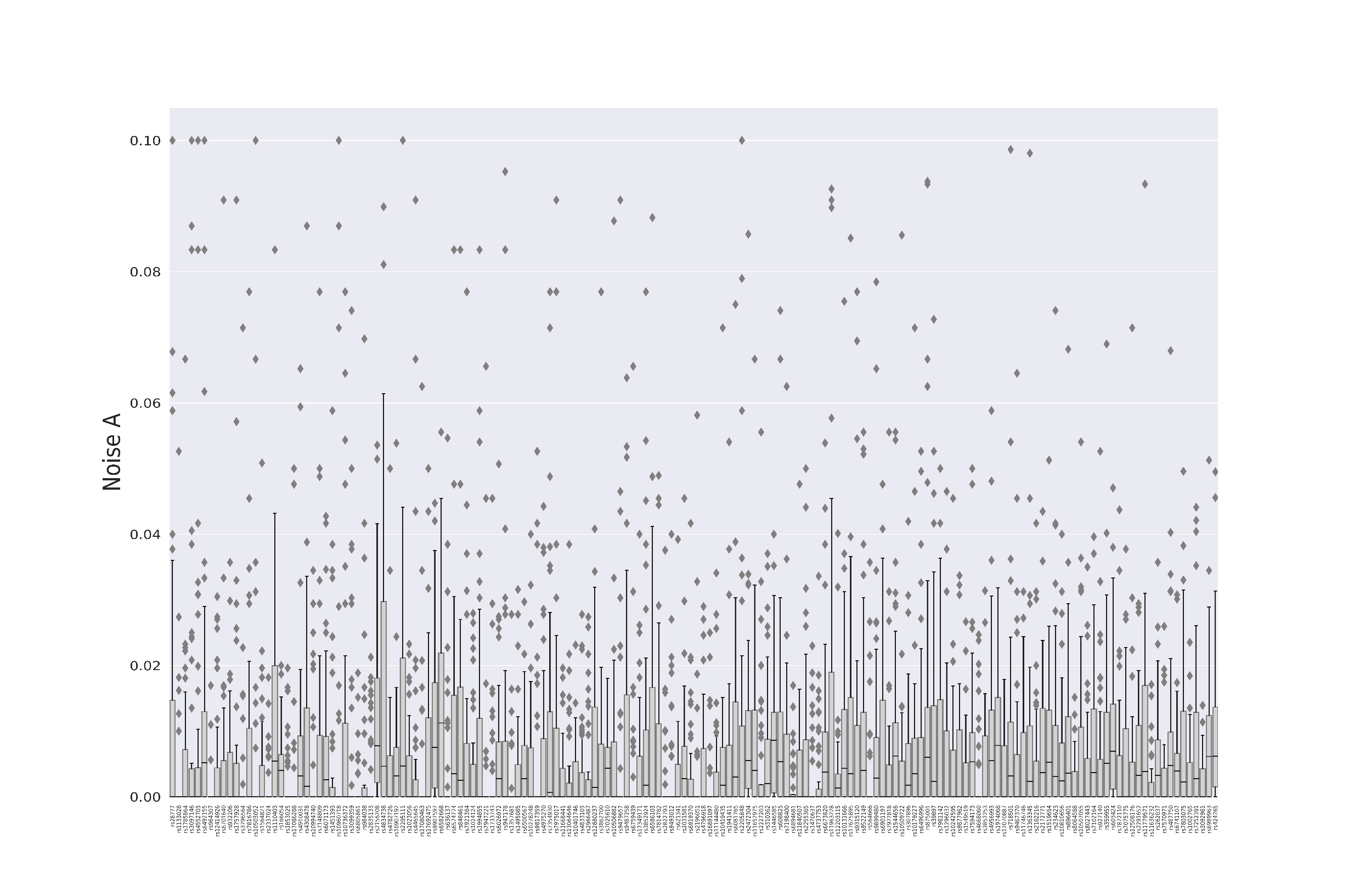


Fig. S3: % noise for the 50 Syrians sequenced for the 164 SNPs included in the QIAseq assay. Noise was estimated as the number of reads that were different from the called genotype divided by the total number of reads for the marker in question. Data were not filtered to allow the direct assessment of the noise.

% Noise

Table S4: Locus drop-out between replicates as well as between UCPH and KCL for the sensitivity study.

| **Amount DNA** | **UCPH Replicate 1** | **UCPH Replicate 2** | **KCL** |
| --- | --- | --- | --- |
| **10 ng** | rs718501 | rs718501 |  |
| **2 ng** |  | rs718501 |  |
| **0.5 ng** | rs1363345, rs1470637, rs1733743, rs3796564, rs4552703, rs4956993, rs7570971, rs7814782, rs7975017, rs9315120, rs9487258 | rs10401746, rs10506882, rs1110403, rs12508176, rs12880237, rs13064646, rs1470637, rs1544656, rs1733743, rs17625895, rs1941411, rs1974068, rs2196051, rs2205848, rs2966487, rs39897, rs6071173, rs6492155, rs8027443, rs927140, rs947178, rs9493012, rs984038 | rs718501, rs896401, rs11746746, rs6989963, rs7102164, rs7872110,  rs359955 |
| **0.25 ng** | rs1002765, rs10278248, rs10907192, rs11779571, rs12880237, rs17086288, rs1733743, rs1785864, rs1941411, rs1994855, rs2196051, rs3796564, rs4465645, rs4796918, rs4833103, rs6026972, rs6574774, rs6901197, rs6919370, rs6989963, rs7075775, rs896401,  rs927140, rs9522149,  rs964307, rs9817359, rs984038 | rs1024124, rs10736372, rs10960718, rs11636232, rs11746746, rs11840507, rs11963228, rs12203115, rs12508176, rs12880237, rs13064646, rs1470637, rs1733743, rs17692475, rs2172771, rs2182216, rs2196051, rs2205848, rs2227203, rs262037, rs2822793, rs2835133, rs2966487, rs3099359, rs3796564, rs3852924, rs424765, rs4465645, rs4552703, rs4782726, rs4833103, rs6026972, rs6071173, rs621341, rs636137, rs6496996, rs6500567, rs6759439, rs6894681, rs6901197, rs7025610, rs7102164, rs7148809, rs718501, rs7354930, rs7594173, rs7814782, rs7872110, rs7921384, rs7922818, rs7975017, rs8027443, rs8064588, rs848461, rs875001, rs896401, rs9487258, rs9493012, rs9522149, rs964307, rs984038, rs9877982 | rs10503052, rs11652975, rs1470637, rs17080463, rs17086288, rs1733743, rs17692475, rs2835133, rs3862700, rs4834738, rs603424, rs6582668, rs6894681, rs7947221, rs9522149, rs9907597, rs9315120, rs6901197, rs310362, rs424765,  rs6805861, rs10507055, rs964307, rs718501, rs11963228, rs17625895, rs1002765, rs896401,  rs4552703 |
| **0.125 ng** | rs1002765, rs10131666, rs10179227, rs1023056, rs1024124, rs10242595, rs10278248, rs10401746, rs10419435, rs10503052, rs10507055, rs10509722, rs10736372, rs10810656, rs10907192, rs10960718, rs10994740, rs11144480, rs1133028, rs11652975, rs11668441, rs11746746, rs11779571, rs11840507, rs11963228, rs12203115, rs12414926, rs12508176, rs12880237, rs1296032, rs13064646, rs1357681, rs1363345, rs1446585, rs1451393, rs1470637, rs1519654, rs1544656, rs1549519, rs166054, rs16891097, rs17080463, rs17086288, rs1733743, rs17349171, rs1757928, rs17625895, rs1785864, rs1941411, rs1974068, rs1994855, rs2172771, rs2196051, rs2205848, rs2227203, rs2255365, rs2291652, rs2295111, rs2337024, rs234623, rs2472304, rs262037, rs2822793, rs2835133,  rs28777, rs2966487,  rs307896, rs3092981, rs3097146, rs3099359, rs310362, rs359955,  rs3796564, rs3852253, rs3862700, rs39897,  rs424765, rs4308478, rs4465645, rs4552703, rs4668060, rs4737753, rs4782726, rs4796918, rs4833103, rs4834738, rs487750, rs4910031, rs4956993, rs4975270, rs564666, rs6026972,  rs603424, rs6071173, rs6081765, rs608825,  rs621341, rs636137,  rs6492155, rs6496996, rs6500567, rs6574774, rs6582668, rs6701640, rs6741107, rs6759439, rs6805861, rs6894681, rs6901197, rs6919370, rs6989963, rs7025610, rs7075775, rs7148809, rs718501, rs7198400, rs7252391, rs7354930, rs7570971, rs7594173, rs7803075, rs7814782, rs7872110, rs7921384, rs7922818, rs7947221, rs7975017, rs7981142, rs8027443, rs8064588, rs848461, rs875001,  rs896401, rs927140,  rs9315120, rs932206, rs9467370, rs9479657, rs9487258, rs9493012, rs9522149, rs964307, rs9817359, rs984038, rs9877982, rs9899480, rs9907597 | rs10179227, rs1023056, rs1024124, rs10401746, rs10419435, rs10503052, rs10506882, rs10507055, rs10509722, rs10736372, rs10810656, rs10907192, rs11144480, rs1133028, rs11652975, rs11779571, rs11840507, rs11963228, rs12203115, rs12414926, rs12880237, rs1296032, rs13064646, rs1363345, rs1446585, rs1451393, rs1470637, rs1495085, rs1519654, rs1549519, rs1564821, rs166054, rs16891097, rs17086288, rs1733743, rs17625895, rs17692475, rs1785864, rs1853025, rs1974068, rs1994855, rs2031581, rs2156208, rs2172771, rs2182216, rs2196051, rs2205848, rs2227203, rs2255365, rs2295111, rs2337024, rs234623,  rs262037, rs2822793, rs2835133, rs28777, rs2966487, rs3092981, rs3097146, rs3099359, rs359955, rs3796564, rs3852253, rs3862700, rs4308478, rs4552703, rs4737753, rs4782726, rs4796918, rs4834738, rs4910031, rs4956993, rs4975270, rs564666, rs6026972, rs603424, rs6071173, rs6492155, rs6500567, rs6574774, rs6582668, rs6759439, rs6894681, rs6901197, rs6919370, rs6989963, rs7075775, rs7102164, rs7148809, rs718501, rs7198400, rs7252391, rs7354930, rs7570971, rs7803075, rs7814782, rs7816786, rs7872110, rs7922818, rs7975017, rs7981142, rs8027443, rs896401, rs9315120, rs932206, rs9467370, rs947178, rs964307, rs984038, rs9877982, rs9899480 | rs1002765, rs10179227, rs1023056, rs10419435, rs10503052, rs10506882, rs10507055, rs10509722, rs10810656, rs11636232, rs11668441, rs11746746, rs11840507, rs12203115, rs1470637, rs1519654, rs1549519, rs16891097, rs17080463, rs1757928, rs17625895, rs17692475, rs1785864, rs1974068, rs2156208, rs2172771, rs2205848, rs2227203, rs2255365, rs2295111, rs2822793, rs2966487, rs307896, rs3092981, rs3796564, rs3852253, rs3852924, rs4668060, rs4796918, rs4834738, rs4975270, rs564666, rs6026972, rs621341,  rs636137, rs6500567, rs6574774, rs6582668, rs6701640, rs6894681, rs6901197, rs6919370, rs7148809, rs718501, rs7198400, rs7252391, rs7872110, rs7975017, rs896401, rs932206,  rs964307, rs9817359, rs10278248, rs12880237 |

Table S5: Number of allele drop-outs for the sensitivity study. A total of 46 of 164 loci had allele drop-outs.

| **Locus** | **No. of allele drop-outs** |
| --- | --- |
| rs4782726 | 5 |
| rs1941411 | 4 |
| rs4956993 | 4 |
| rs718501 | 4 |
| rs9907597 | 4 |
| rs1002765 | 3 |
| rs1024124 | 3 |
| rs10278248 | 3 |
| rs1544656 | 3 |
| rs17349171 | 3 |
| rs2156208 | 3 |
| rs6989963 | 3 |
| rs7102164 | 3 |
| rs7814782 | 3 |
| rs10506882 | 2 |
| rs10960718 | 2 |
| rs11652975 | 2 |
| rs11963228 | 2 |
| rs1519654 | 2 |
| rs1785864 | 2 |
| rs1974068 | 2 |
| rs1994855 | 2 |
| rs2196051 | 2 |
| rs6071173 | 2 |
| rs636137 | 2 |
| rs7803075 | 2 |

Table S5 continued

| **Locus** | **No. of allele drop-outs** |
| --- | --- |
| rs7872110 | 2 |
| rs848461 | 2 |
| rs9487258 | 2 |
| rs11746746 | 1 |
| rs12203115 | 1 |
| rs12880237 | 1 |
| rs1363345 | 1 |
| rs1564821 | 1 |
| rs1733743 | 1 |
| rs3092981 | 1 |
| rs310362 | 1 |
| rs3796564 | 1 |
| rs424765 | 1 |
| rs6026972 | 1 |
| rs6496996 | 1 |
| rs6919370 | 1 |
| rs7148809 | 1 |
| rs896401 | 1 |
| rs927140 | 1 |
| rs9522149 | 1 |


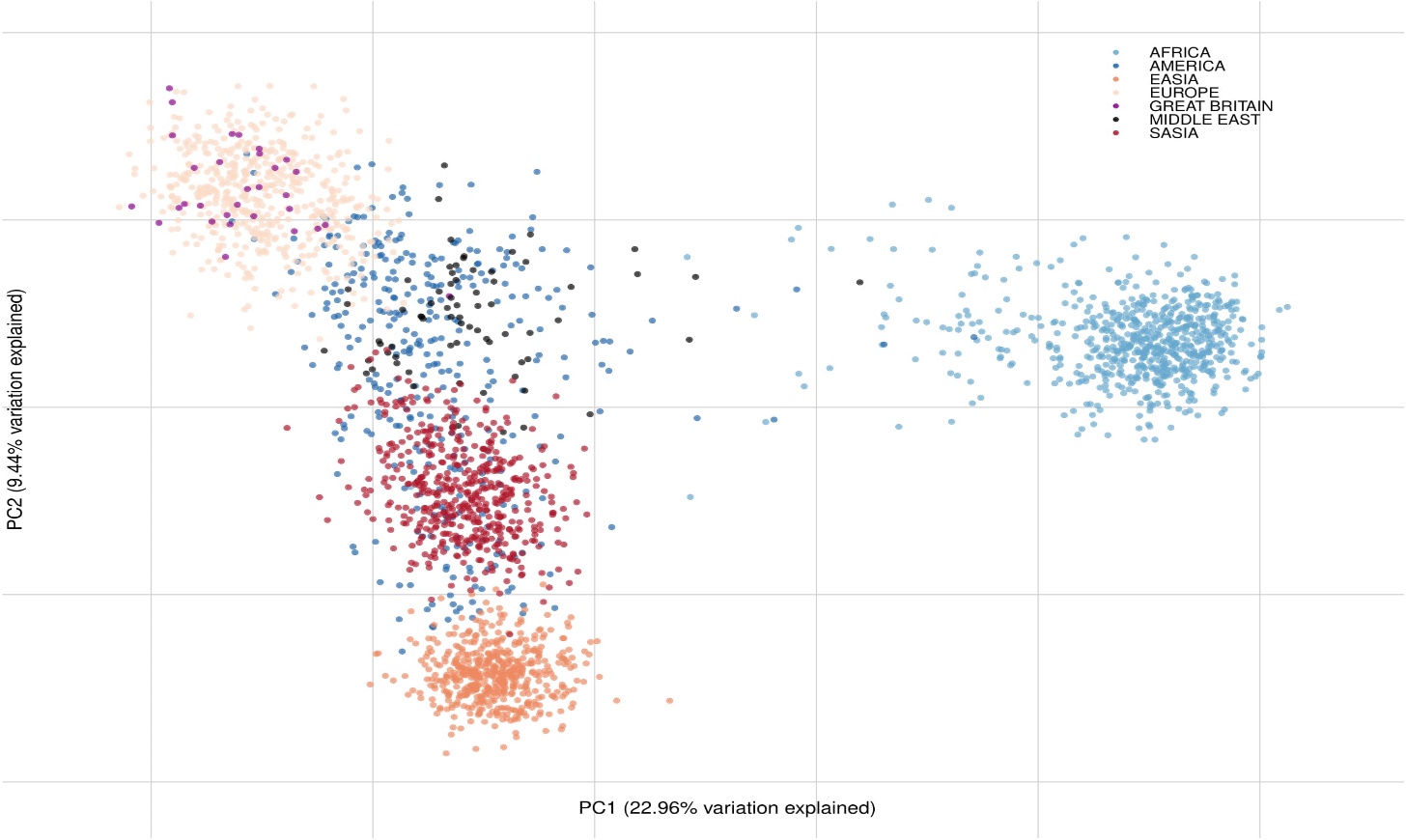


Figure S4: PCA plot of the studied populations and 1000 Genomes reference data. Meta-populations are listed in Table S2. ‘EASIA’ refers to East Asia and ‘SASIA’ refers to South-Central Asia. The individuals from Great Britain typed in this study are highlighted in purple.


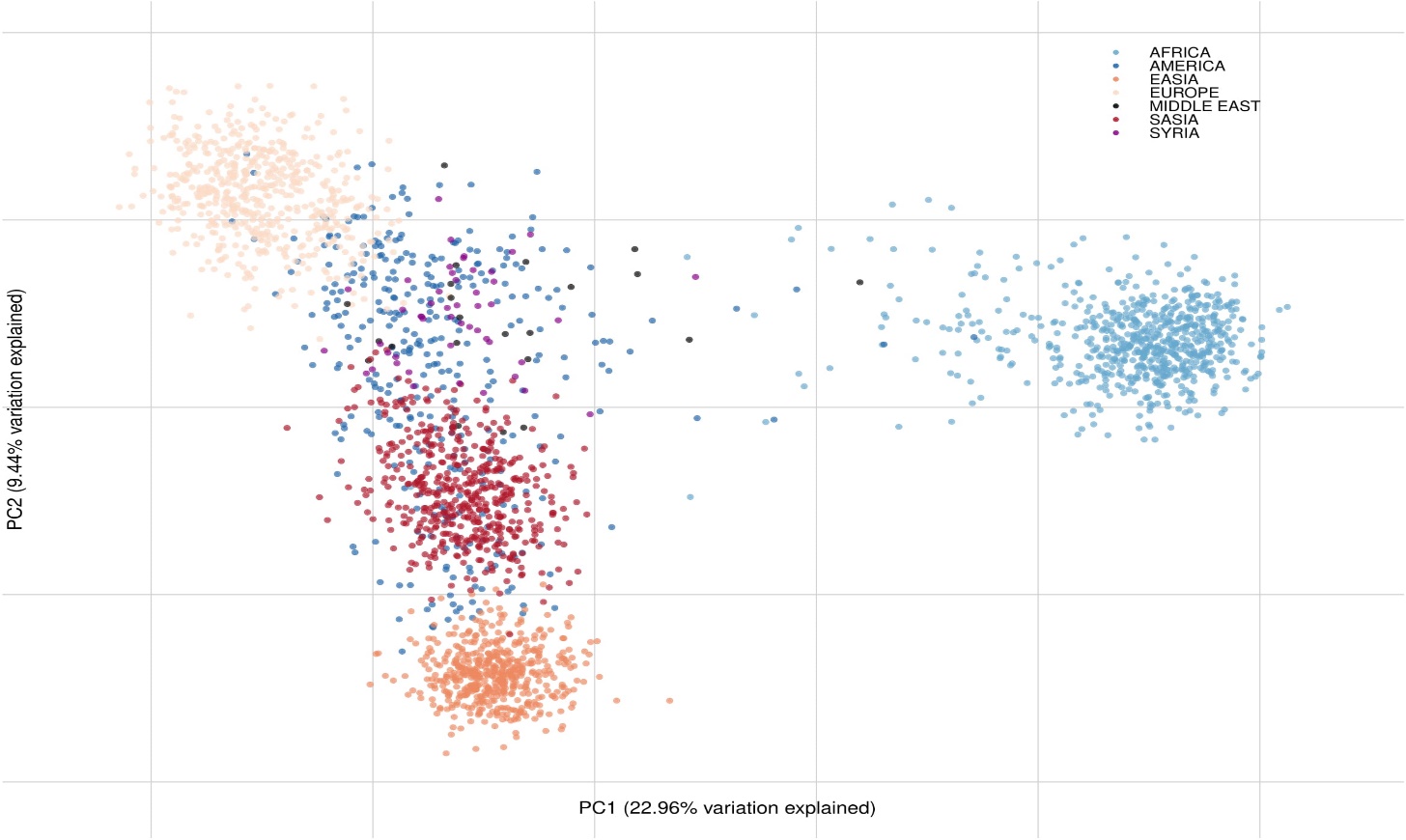


Figure S5: PCA plot of the studied populations and 1000 Genomes reference data. Meta-populations are listed in Table S2. ‘EASIA’ refers to East Asia and ‘SASIA’ refers to South-Central Asia. The individuals from Syria are highlighted in purple, and the individuals from the Middle East are highlighted in black.

Figure S6. Electrophoresis of libraries with 2 ng DNA input analysed with the 2100 Bioanalyzer. a) Library generated according to the protocol without dilution of adapters. Adapter dimers were observed at approx. 200 base pairs (bp). b) Library generated with 10x diluted adapters.

a


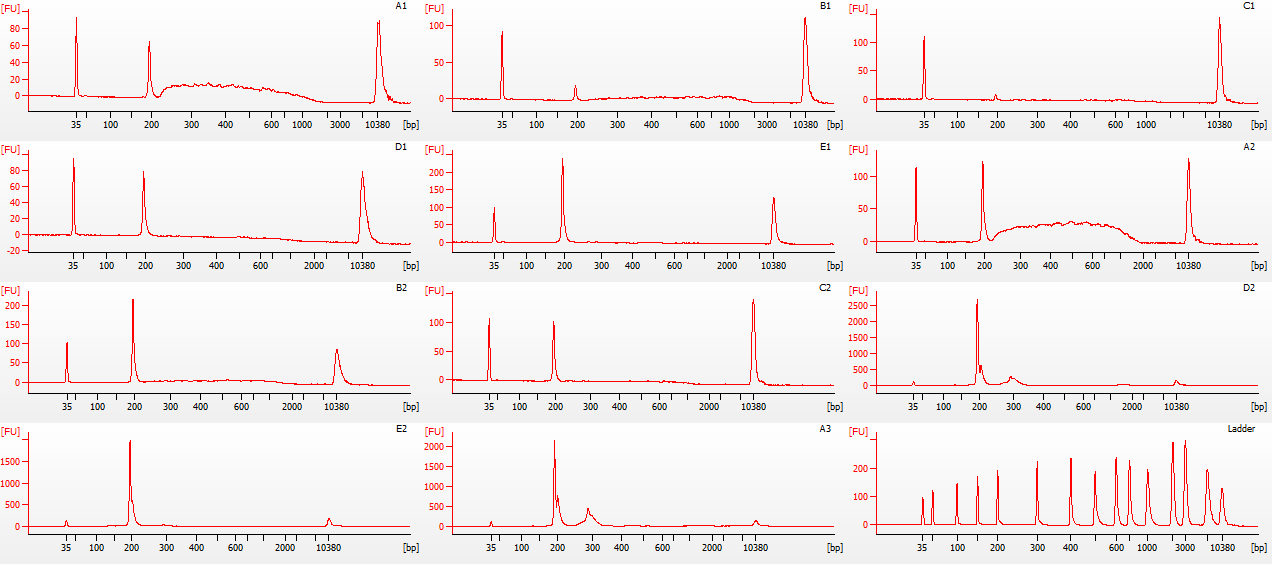


b


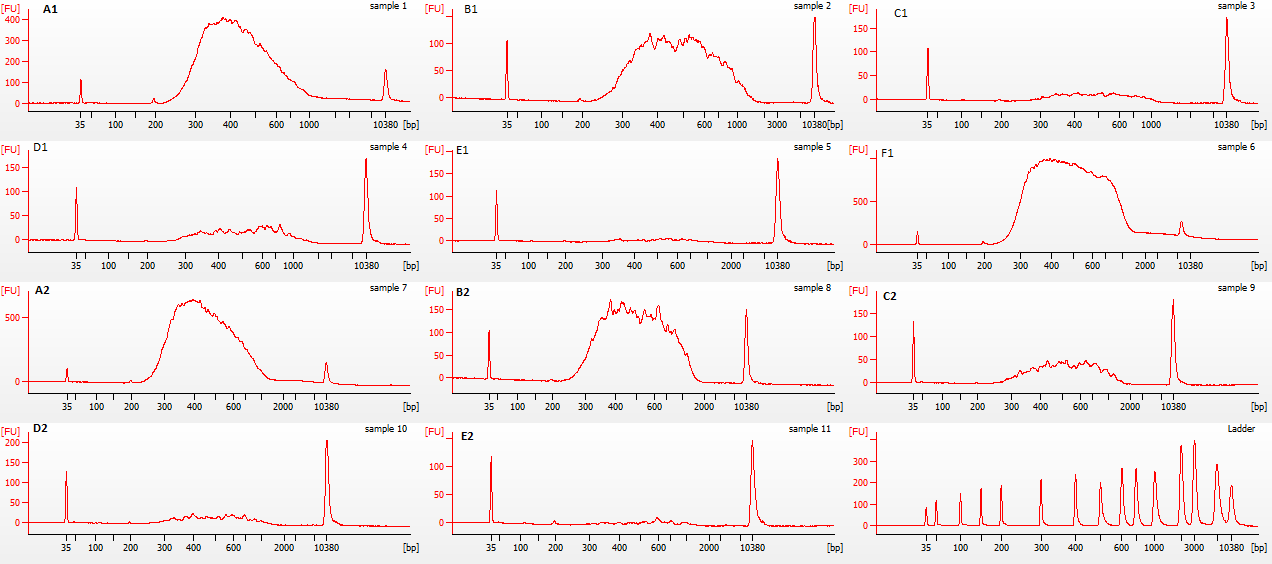


Table S6: Number of UMI reads per locus in the sensitivity study. The dilution series was run in duplicate in UCPH and in KCL, it was sequenced once.

|  | **UCPH** | | | | | | | | | | | | **KCL** | | | | |
| --- | --- | --- | --- | --- | --- | --- | --- | --- | --- | --- | --- | --- | --- | --- | --- | --- | --- |
|  | **Replicate 1** | | | | | | **Replicate 2** | | | | | |  | | | | |
| **Locus** | **40 ng** | **10 ng** | **2 ng** | **0.5 ng** | **0.25 ng** | **0.125 ng** | **40 ng** | **10 ng** | **2 ng** | **0.5 ng** | **0.25 ng** | **0.125 ng** | **10 ng** | **2 ng** | **0.5 ng** | **0.25 ng** | **0.125 ng** |
| rs1002765 | 1028 | 682 | 438 | 54 | 13 | 2 | 1205 | 494 | 253 | 36 | 42 | 25 | 760 | 1201 | 1074 | 605 | 3 |
| rs10131666 | 1121 | 657 | 360 | 133 | 139 | 12 | 1217 | 540 | 314 | 36 | 86 | 20 | 876 | 1381 | 845 | 317 | 351 |
| rs10179227 | 919 | 798 | 306 | 59 | 80 | 11 | 1177 | 444 | 313 | 33 | 43 | 7 | 632 | 937 | 664 | 86 | 1 |
| rs1023056 | 945 | 705 | 361 | 60 | 29 | 6 | 991 | 511 | 267 | 87 | 92 | 2 | 610 | 936 | 561 | 261 | 1 |
| rs1024124 | 1009 | 767 | 350 | 120 | 37 | 1 | 1215 | 583 | 215 | 30 | 2 | 16 | 900 | 2078 | 1732 | 180 | 100 |
| rs10242595 | 1325 | 796 | 382 | 47 | 87 | 1 | 1348 | 565 | 347 | 31 | 55 | 59 | 733 | 1575 | 425 | 33 | 26 |
| rs10278248 | 1244 | 1190 | 408 | 169 | 79 | 0 | 1501 | 656 | 338 | 72 | 52 | 20 | 1054 | 1268 | 825 | 226 | 479 |
| rs10401746 | 1106 | 605 | 332 | 92 | 70 | 1 | 1184 | 482 | 273 | 19 | 43 | 14 | 893 | 938 | 1044 | 232 | 666 |
| rs10419435 | 1038 | 690 | 331 | 126 | 90 | 0 | 1075 | 479 | 248 | 50 | 44 | 14 | 1052 | 1178 | 1126 | 445 | 0 |
| rs10503052 | 1129 | 784 | 548 | 52 | 71 | 0 | 1290 | 602 | 222 | 178 | 61 | 1 | 777 | 1179 | 1094 | 1 | 0 |
| rs10506882 | 880 | 671 | 389 | 84 | 51 | 22 | 1127 | 470 | 262 | 41 | 43 | 0 | 457 | 858 | 697 | 128 | 2 |
| rs10507055 | 1109 | 714 | 474 | 186 | 131 | 1 | 1199 | 493 | 271 | 32 | 33 | 7 | 618 | 1046 | 523 | 18 | 0 |
| rs10509722 | 1017 | 669 | 471 | 43 | 65 | 2 | 1151 | 546 | 345 | 26 | 30 | 1 | 954 | 1312 | 858 | 127 | 4 |
| rs10736372 | 879 | 964 | 601 | 94 | 93 | 9 | 1114 | 550 | 327 | 62 | 6 | 3 | 766 | 1224 | 191 | 215 | 121 |
| rs10810656 | 998 | 700 | 324 | 95 | 169 | 12 | 1100 | 526 | 250 | 37 | 23 | 0 | 898 | 1516 | 823 | 87 | 0 |
| rs10907192 | 1074 | 797 | 372 | 155 | 18 | 11 | 1217 | 545 | 446 | 45 | 20 | 3 | 582 | 1052 | 632 | 347 | 126 |
| rs10960718 | 939 | 629 | 338 | 108 | 64 | 5 | 982 | 477 | 222 | 46 | 2 | 40 | 680 | 1100 | 877 | 375 | 358 |
| rs10994740 | 954 | 846 | 377 | 193 | 69 | 1 | 1188 | 472 | 161 | 34 | 46 | 42 | 783 | 972 | 652 | 149 | 55 |
| rs1110403 | 1086 | 541 | 327 | 95 | 55 | 20 | 1263 | 533 | 379 | 16 | 47 | 20 | 1091 | 1906 | 2330 | 223 | 485 |
| rs11144480 | 1064 | 761 | 393 | 206 | 32 | 12 | 1278 | 543 | 494 | 57 | 50 | 1 | 887 | 1870 | 1281 | 181 | 333 |
| rs1133028 | 982 | 855 | 408 | 110 | 84 | 8 | 1181 | 493 | 264 | 37 | 77 | 11 | 646 | 1057 | 1064 | 164 | 563 |
| rs11636232 | 1093 | 912 | 405 | 164 | 132 | 34 | 1286 | 549 | 243 | 37 | 3 | 35 | 946 | 1174 | 591 | 90 | 6 |
| rs11652975 | 1287 | 867 | 365 | 167 | 88 | 1 | 1338 | 549 | 385 | 84 | 29 | 92 | 913 | 1460 | 801 | 4 | 238 |
| rs11668441 | 1031 | 797 | 327 | 27 | 35 | 5 | 1050 | 571 | 292 | 43 | 32 | 99 | 812 | 1291 | 1282 | 225 | 0 |
| rs11746746 | 1087 | 694 | 394 | 119 | 43 | 13 | 1295 | 524 | 348 | 44 | 19 | 47 | 779 | 1001 | 210 | 111 | 1 |

Table S6 continued

|  | **UCPH** | | | | | | | | | | | | **KCL** | | | | |
| --- | --- | --- | --- | --- | --- | --- | --- | --- | --- | --- | --- | --- | --- | --- | --- | --- | --- |
|  | **Replicate 1** | | | | | | **Replicate 2** | | | | | |  | | | | |
| **Locus** | **40 ng** | **10 ng** | **2 ng** | **0.5 ng** | **0.25 ng** | **0.125 ng** | **40 ng** | **10 ng** | **2 ng** | **0.5 ng** | **0.25 ng** | **0.125 ng** | **10 ng** | **2 ng** | **0.5 ng** | **0.25 ng** | **0.125 ng** |
| rs11779571 | 1209 | 746 | 313 | 131 | 17 | 9 | 1246 | 550 | 611 | 86 | 107 | 7 | 873 | 1654 | 1656 | 121 | 68 |
| rs11840507 | 900 | 687 | 383 | 148 | 68 | 7 | 1032 | 445 | 390 | 25 | 18 | 14 | 622 | 1261 | 575 | 80 | 0 |
| rs11963228 | 1182 | 902 | 516 | 175 | 73 | 1 | 1391 | 636 | 444 | 68 | 50 | 16 | 974 | 1341 | 1317 | 496 | 238 |
| rs12070887 | 1050 | 732 | 395 | 96 | 87 | 25 | 1134 | 514 | 245 | 49 | 45 | 29 | 945 | 1253 | 585 | 93 | 126 |
| rs12203115 | 1787 | 1297 | 614 | 134 | 184 | 0 | 1815 | 871 | 411 | 88 | 28 | 0 | 952 | 1362 | 819 | 391 | 4 |
| rs12414926 | 896 | 710 | 355 | 30 | 106 | 12 | 973 | 440 | 185 | 23 | 76 | 1 | 751 | 1061 | 703 | 206 | 41 |
| rs12508176 | 1073 | 908 | 245 | 24 | 35 | 0 | 1092 | 561 | 481 | 6 | 0 | 20 | 566 | 910 | 771 | 25 | 191 |
| rs12880237 | 778 | 563 | 274 | 48 | 7 | 0 | 656 | 296 | 139 | 13 | 63 | 11 | 290 | 474 | 282 | 52 | 141 |
| rs1296032 | 958 | 750 | 342 | 37 | 60 | 2 | 1123 | 465 | 298 | 31 | 52 | 1 | 615 | 1237 | 992 | 39 | 278 |
| rs13064646 | 933 | 798 | 524 | 66 | 156 | 3 | 1140 | 530 | 240 | 18 | 3 | 0 | 790 | 1161 | 864 | 53 | 52 |
| rs1357681 | 1186 | 1003 | 451 | 157 | 88 | 2 | 1409 | 637 | 367 | 42 | 43 | 28 | 903 | 1805 | 1689 | 161 | 256 |
| rs1363345 | 1148 | 823 | 413 | 134 | 234 | 1 | 1379 | 538 | 255 | 88 | 54 | 1 | 1088 | 1895 | 1357 | 305 | 140 |
| rs1446585 | 933 | 698 | 415 | 57 | 87 | 2 | 964 | 404 | 196 | 89 | 33 | 1 | 567 | 865 | 646 | 48 | 189 |
| rs1451393 | 893 | 610 | 350 | 85 | 86 | 4 | 850 | 465 | 290 | 35 | 62 | 0 | 536 | 959 | 871 | 100 | 211 |
| rs1470637 | 535 | 323 | 100 | 8 | 21 | 1 | 605 | 272 | 103 | 11 | 6 | 0 | 89 | 152 | 75 | 0 | 0 |
| rs1495085 | 1086 | 848 | 555 | 180 | 78 | 23 | 1140 | 505 | 401 | 66 | 60 | 19 | 1040 | 1530 | 1584 | 215 | 270 |
| rs1519654 | 1025 | 607 | 261 | 96 | 27 | 2 | 1151 | 455 | 249 | 39 | 39 | 7 | 707 | 950 | 909 | 500 | 2 |
| rs1544656 | 843 | 583 | 245 | 76 | 115 | 3 | 844 | 357 | 190 | 75 | 69 | 51 | 539 | 850 | 478 | 133 | 103 |
| rs1549519 | 986 | 744 | 386 | 138 | 129 | 5 | 1150 | 472 | 193 | 57 | 23 | 5 | 854 | 1304 | 1136 | 175 | 2 |
| rs1564821 | 1182 | 784 | 422 | 126 | 127 | 27 | 1393 | 565 | 294 | 20 | 81 | 4 | 845 | 1445 | 577 | 219 | 86 |
| rs166054 | 1226 | 780 | 486 | 93 | 120 | 13 | 1474 | 631 | 446 | 39 | 57 | 1 | 1084 | 1836 | 1068 | 654 | 109 |
| rs16891097 | 988 | 669 | 440 | 53 | 101 | 13 | 983 | 498 | 238 | 34 | 79 | 2 | 585 | 1184 | 907 | 44 | 0 |
| rs17080463 | 1156 | 898 | 404 | 89 | 48 | 3 | 1424 | 618 | 215 | 38 | 31 | 27 | 829 | 1101 | 1335 | 0 | 1 |
| rs17086288 | 1210 | 969 | 449 | 46 | 19 | 2 | 1447 | 517 | 453 | 61 | 38 | 1 | 922 | 1413 | 1328 | 0 | 98 |
| rs1733743 | 1018 | 825 | 421 | 58 | 11 | 14 | 1188 | 510 | 447 | 33 | 13 | 0 | 713 | 1053 | 498 | 0 | 78 |
| rs17349171 | 973 | 815 | 337 | 144 | 28 | 19 | 1131 | 427 | 365 | 42 | 33 | 23 | 676 | 972 | 793 | 179 | 480 |

Table S6 continued

|  | **UCPH** | | | | | | | | | | | | **KCL** | | | | |
| --- | --- | --- | --- | --- | --- | --- | --- | --- | --- | --- | --- | --- | --- | --- | --- | --- | --- |
|  | **Replicate 1** | | | | | | **Replicate 2** | | | | | |  |  |  |  |  |
| **Locus** | **40 ng** | **10 ng** | **2 ng** | **0.5 ng** | **0.25 ng** | **0.125 ng** | **40 ng** | **10 ng** | **2 ng** | **0.5 ng** | **0.25 ng** | **0.125 ng** | **10 ng** | **2 ng** | **0.5 ng** | **0.25 ng** | **0.125 ng** |
| rs1757928 | 1609 | 1271 | 606 | 156 | 82 | 2 | 1838 | 716 | 411 | 45 | 50 | 35 | 658 | 1168 | 525 | 31 | 2 |
| rs17625895 | 1012 | 532 | 319 | 36 | 45 | 11 | 991 | 451 | 355 | 37 | 65 | 0 | 600 | 727 | 563 | 192 | 1 |
| rs17692475 | 951 | 758 | 407 | 82 | 82 | 22 | 1135 | 398 | 417 | 57 | 1 | 19 | 912 | 1128 | 1603 | 9 | 5 |
| rs1785864 | 746 | 757 | 314 | 127 | 36 | 13 | 886 | 341 | 280 | 23 | 46 | 1 | 535 | 808 | 254 | 31 | 2 |
| rs1853025 | 1180 | 814 | 376 | 108 | 71 | 28 | 1379 | 523 | 322 | 32 | 20 | 7 | 938 | 1235 | 1288 | 217 | 320 |
| rs1941411 | 980 | 759 | 350 | 148 | 52 | 0 | 1208 | 529 | 234 | 37 | 20 | 36 | 755 | 1262 | 795 | 51 | 349 |
| rs1974068 | 978 | 936 | 323 | 120 | 43 | 17 | 1200 | 550 | 331 | 55 | 27 | 2 | 869 | 1538 | 853 | 217 | 0 |
| rs1994855 | 1140 | 867 | 530 | 36 | 267 | 8 | 1442 | 660 | 593 | 60 | 66 | 15 | 1023 | 955 | 699 | 279 | 177 |
| rs2031581 | 922 | 699 | 252 | 89 | 78 | 33 | 1087 | 444 | 220 | 40 | 57 | 3 | 720 | 1080 | 1543 | 193 | 112 |
| rs2156208 | 1184 | 810 | 370 | 227 | 267 | 31 | 1478 | 577 | 242 | 28 | 48 | 14 | 1186 | 1998 | 2000 | 130 | 8 |
| rs2172771 | 1009 | 488 | 272 | 35 | 29 | 4 | 1019 | 440 | 194 | 47 | 5 | 12 | 869 | 1303 | 688 | 137 | 2 |
| rs2182216 | 1093 | 630 | 304 | 132 | 131 | 30 | 1026 | 462 | 324 | 38 | 2 | 13 | 736 | 1428 | 535 | 135 | 40 |
| rs2196051 | 738 | 628 | 272 | 53 | 29 | 0 | 764 | 361 | 112 | 43 | 27 | 1 | 275 | 468 | 482 | 124 | 22 |
| rs2205848 | 1082 | 805 | 380 | 102 | 154 | 0 | 1087 | 533 | 417 | 14 | 0 | 2 | 860 | 1752 | 1013 | 383 | 2 |
| rs2227203 | 970 | 738 | 603 | 97 | 121 | 0 | 949 | 419 | 313 | 44 | 11 | 7 | 727 | 1167 | 1064 | 330 | 1 |
| rs2255365 | 1110 | 695 | 320 | 72 | 97 | 6 | 1247 | 479 | 304 | 46 | 40 | 10 | 713 | 1192 | 590 | 306 | 0 |
| rs2291652 | 1128 | 718 | 417 | 86 | 39 | 7 | 1338 | 453 | 385 | 132 | 118 | 39 | 1085 | 1555 | 1667 | 325 | 228 |
| rs2295111 | 1049 | 826 | 574 | 51 | 147 | 16 | 1207 | 500 | 475 | 93 | 91 | 4 | 1144 | 1486 | 1265 | 141 | 0 |
| rs2337024 | 1006 | 715 | 414 | 43 | 73 | 7 | 1099 | 444 | 286 | 59 | 20 | 18 | 744 | 1473 | 1577 | 90 | 143 |
| rs234623 | 1374 | 881 | 471 | 76 | 29 | 12 | 1440 | 667 | 256 | 56 | 136 | 1 | 1036 | 1197 | 1018 | 165 | 183 |
| rs2472304 | 1939 | 1128 | 597 | 111 | 193 | 3 | 2149 | 833 | 535 | 93 | 102 | 41 | 1226 | 1941 | 2427 | 402 | 22 |
| rs262037 | 963 | 642 | 365 | 94 | 39 | 2 | 1097 | 552 | 234 | 72 | 0 | 0 | 579 | 799 | 513 | 100 | 197 |
| rs2822793 | 1170 | 940 | 465 | 75 | 63 | 2 | 1271 | 552 | 278 | 57 | 5 | 8 | 788 | 922 | 778 | 288 | 2 |
| rs2835133 | 855 | 572 | 324 | 70 | 78 | 1 | 1008 | 350 | 199 | 27 | 7 | 16 | 399 | 861 | 661 | 2 | 144 |
| rs28777 | 944 | 635 | 273 | 80 | 29 | 5 | 959 | 400 | 168 | 76 | 95 | 1 | 977 | 1331 | 1223 | 199 | 247 |

Table S6 continued

|  | **UCPH** | | | | | | | | | | | | **KCL** | | | | |
| --- | --- | --- | --- | --- | --- | --- | --- | --- | --- | --- | --- | --- | --- | --- | --- | --- | --- |
|  | **Replicate 1** | | | | | | **Replicate 2** | | | | | |  |  |  |  |  |
| **Locus** | **40 ng** | **10 ng** | **2 ng** | **0.5 ng** | **0.25 ng** | **0.125 ng** | **40 ng** | **10 ng** | **2 ng** | **0.5 ng** | **0.25 ng** | **0.125 ng** | **10 ng** | **2 ng** | **0.5 ng** | **0.25 ng** | **0.125 ng** |
| rs2966487 | 1217 | 1224 | 405 | 89 | 64 | 0 | 1495 | 567 | 250 | 5 | 33 | 7 | 702 | 1234 | 628 | 265 | 7 |
| rs307896 | 1200 | 852 | 509 | 122 | 54 | 13 | 1353 | 528 | 267 | 49 | 128 | 37 | 1041 | 2073 | 1087 | 155 | 1 |
| rs3092981 | 867 | 531 | 203 | 95 | 43 | 0 | 901 | 401 | 216 | 38 | 48 | 49 | 549 | 704 | 873 | 200 | 1 |
| rs3097146 | 1162 | 1010 | 612 | 146 | 70 | 12 | 1363 | 540 | 476 | 70 | 58 | 17 | 1050 | 1247 | 428 | 76 | 102 |
| rs3099359 | 1084 | 851 | 505 | 152 | 81 | 0 | 1385 | 512 | 197 | 65 | 2 | 16 | 1035 | 1400 | 704 | 620 | 212 |
| rs310362 | 1063 | 789 | 451 | 125 | 92 | 0 | 1190 | 603 | 351 | 53 | 33 | 47 | 866 | 1598 | 744 | 381 | 384 |
| rs359955 | 1548 | 1467 | 636 | 294 | 214 | 1 | 1925 | 820 | 491 | 124 | 129 | 5 | 1232 | 1974 | 972 | 176 | 260 |
| rs3796564 | 943 | 655 | 345 | 128 | 137 | 1 | 968 | 399 | 236 | 27 | 138 | 6 | 814 | 1316 | 1200 | 377 | 2 |
| rs3852253 | 1292 | 1248 | 585 | 60 | 48 | 16 | 1817 | 756 | 461 | 22 | 32 | 2 | 856 | 1259 | 1111 | 145 | 1 |
| rs3852924 | 1250 | 1296 | 391 | 150 | 90 | 27 | 1569 | 790 | 538 | 87 | 15 | 23 | 1425 | 2294 | 618 | 110 | 4 |
| rs3862700 | 974 | 878 | 485 | 72 | 60 | 1 | 1247 | 501 | 369 | 68 | 32 | 1 | 761 | 946 | 631 | 4 | 102 |
| rs39897 | 1064 | 681 | 447 | 108 | 77 | 18 | 1043 | 484 | 354 | 10 | 43 | 20 | 984 | 1671 | 1483 | 544 | 216 |
| rs424765 | 1271 | 886 | 573 | 138 | 24 | 8 | 1623 | 613 | 486 | 63 | 123 | 70 | 1294 | 1628 | 2586 | 11 | 403 |
| rs4308478 | 1016 | 702 | 460 | 138 | 91 | 11 | 1303 | 482 | 305 | 36 | 96 | 3 | 962 | 2017 | 1194 | 519 | 437 |
| rs4465645 | 925 | 835 | 420 | 83 | 17 | 1 | 1164 | 483 | 377 | 39 | 16 | 31 | 1028 | 1812 | 1201 | 276 | 62 |
| rs4552703 | 1052 | 798 | 383 | 13 | 69 | 12 | 1144 | 497 | 402 | 24 | 10 | 9 | 723 | 1160 | 542 | 285 | 251 |
| rs4668060 | 1156 | 681 | 561 | 127 | 97 | 1 | 1067 | 507 | 424 | 57 | 102 | 37 | 782 | 1286 | 700 | 106 | 3 |
| rs4737753 | 1134 | 1010 | 449 | 218 | 21 | 11 | 1347 | 556 | 234 | 24 | 65 | 2 | 846 | 1148 | 348 | 354 | 310 |
| rs4782726 | 1203 | 924 | 471 | 142 | 54 | 5 | 1061 | 492 | 399 | 37 | 3 | 3 | 956 | 1584 | 1405 | 245 | 107 |
| rs4796918 | 1125 | 929 | 396 | 175 | 4 | 6 | 1249 | 541 | 321 | 41 | 27 | 10 | 815 | 864 | 783 | 248 | 1 |
| rs4833103 | 1056 | 600 | 350 | 118 | 10 | 7 | 1103 | 415 | 291 | 64 | 8 | 31 | 750 | 1079 | 1070 | 212 | 99 |
| rs4834738 | 1060 | 772 | 380 | 118 | 28 | 5 | 1169 | 539 | 359 | 21 | 50 | 0 | 913 | 1187 | 872 | 8 | 3 |
| rs487750 | 921 | 584 | 404 | 35 | 179 | 6 | 954 | 363 | 202 | 69 | 60 | 29 | 702 | 873 | 871 | 251 | 475 |
| rs4910031 | 1297 | 1034 | 538 | 110 | 173 | 13 | 1561 | 689 | 451 | 98 | 58 | 0 | 1091 | 1286 | 401 | 254 | 215 |
| rs4956993 | 1018 | 629 | 357 | 47 | 37 | 8 | 983 | 427 | 218 | 36 | 43 | 2 | 653 | 912 | 302 | 30 | 21 |
| rs4975270 | 920 | 562 | 284 | 26 | 141 | 0 | 948 | 351 | 237 | 31 | 34 | 8 | 534 | 763 | 810 | 65 | 0 |

Table S6 continued

|  | **UCPH** | | | | | | | | | | | | **KCL** | | | | |
| --- | --- | --- | --- | --- | --- | --- | --- | --- | --- | --- | --- | --- | --- | --- | --- | --- | --- |
|  | **Replicate 1** | | | | | | **Replicate 2** | | | | | |  |  |  |  |  |
| **Locus** | **40 ng** | **10 ng** | **2 ng** | **0.5 ng** | **0.25 ng** | **0.125 ng** | **40 ng** | **10 ng** | **2 ng** | **0.5 ng** | **0.25 ng** | **0.125 ng** | **10 ng** | **2 ng** | **0.5 ng** | **0.25 ng** | **0.125 ng** |
| rs564666 | 1058 | 739 | 461 | 117 | 59 | 10 | 1157 | 604 | 383 | 60 | 106 | 1 | 998 | 1037 | 1060 | 357 | 2 |
| rs6026972 | 1056 | 775 | 367 | 66 | 11 | 0 | 1351 | 542 | 276 | 34 | 17 | 1 | 926 | 1505 | 968 | 220 | 0 |
| rs603424 | 1214 | 1317 | 479 | 143 | 81 | 8 | 1593 | 636 | 381 | 47 | 73 | 15 | 1225 | 1437 | 1317 | 5 | 57 |
| rs6071173 | 1023 | 725 | 271 | 58 | 29 | 19 | 1047 | 486 | 250 | 46 | 7 | 2 | 704 | 821 | 272 | 118 | 357 |
| rs6081765 | 1167 | 692 | 407 | 67 | 93 | 7 | 1189 | 461 | 535 | 38 | 90 | 25 | 743 | 1122 | 499 | 397 | 93 |
| rs608825 | 861 | 512 | 266 | 120 | 30 | 0 | 918 | 435 | 217 | 30 | 61 | 96 | 510 | 908 | 621 | 161 | 252 |
| rs621341 | 1026 | 723 | 351 | 47 | 79 | 0 | 1215 | 464 | 354 | 66 | 19 | 20 | 854 | 1396 | 1493 | 216 | 1 |
| rs636137 | 916 | 626 | 374 | 130 | 59 | 8 | 974 | 447 | 308 | 27 | 10 | 29 | 628 | 1146 | 553 | 122 | 0 |
| rs6492155 | 1088 | 940 | 437 | 107 | 64 | 2 | 1246 | 571 | 337 | 18 | 36 | 3 | 1175 | 1699 | 1114 | 25 | 229 |
| rs6496996 | 935 | 614 | 295 | 35 | 83 | 6 | 1035 | 408 | 242 | 36 | 31 | 32 | 537 | 734 | 783 | 245 | 85 |
| rs6500567 | 1021 | 689 | 409 | 92 | 22 | 1 | 961 | 434 | 224 | 36 | 13 | 15 | 1000 | 984 | 488 | 113 | 4 |
| rs6574774 | 1084 | 715 | 435 | 105 | 16 | 11 | 1198 | 495 | 408 | 34 | 27 | 9 | 882 | 1192 | 968 | 187 | 0 |
| rs6582668 | 1250 | 1013 | 573 | 85 | 97 | 14 | 1432 | 551 | 367 | 30 | 55 | 12 | 860 | 1371 | 1261 | 0 | 0 |
| rs6586103 | 1119 | 955 | 551 | 39 | 28 | 33 | 1240 | 604 | 381 | 82 | 56 | 50 | 721 | 1347 | 1047 | 73 | 96 |
| rs6673820 | 1165 | 679 | 375 | 140 | 26 | 20 | 1270 | 478 | 205 | 43 | 28 | 22 | 980 | 1128 | 792 | 670 | 238 |
| rs6701640 | 1055 | 880 | 573 | 62 | 77 | 19 | 1174 | 485 | 532 | 51 | 82 | 45 | 851 | 1266 | 956 | 56 | 0 |
| rs6741107 | 1051 | 728 | 343 | 119 | 144 | 0 | 1266 | 461 | 412 | 53 | 21 | 28 | 877 | 1061 | 1599 | 195 | 277 |
| rs6759439 | 851 | 604 | 311 | 32 | 112 | 3 | 1016 | 433 | 220 | 41 | 8 | 8 | 621 | 898 | 941 | 183 | 21 |
| rs6805861 | 1054 | 888 | 433 | 68 | 109 | 7 | 1215 | 559 | 279 | 63 | 120 | 34 | 872 | 1512 | 340 | 12 | 445 |
| rs6894681 | 1094 | 778 | 649 | 73 | 42 | 5 | 1274 | 521 | 426 | 60 | 7 | 6 | 643 | 1109 | 1415 | 0 | 0 |
| rs6901197 | 977 | 839 | 431 | 194 | 44 | 11 | 1128 | 468 | 294 | 45 | 15 | 3 | 830 | 1420 | 918 | 669 | 4 |
| rs6919370 | 803 | 469 | 237 | 78 | 19 | 2 | 881 | 386 | 270 | 38 | 30 | 8 | 471 | 902 | 544 | 123 | 0 |
| rs6989963 | 1316 | 997 | 404 | 83 | 6 | 3 | 1294 | 748 | 480 | 87 | 54 | 1 | 916 | 1512 | 896 | 98 | 102 |
| rs7025610 | 1048 | 703 | 437 | 165 | 229 | 2 | 1226 | 531 | 611 | 34 | 11 | 69 | 952 | 1148 | 1200 | 142 | 326 |
| rs7075775 | 1057 | 643 | 471 | 59 | 18 | 9 | 1183 | 487 | 240 | 31 | 109 | 2 | 573 | 998 | 606 | 156 | 43 |
| rs7102164 | 1112 | 696 | 426 | 94 | 37 | 56 | 1174 | 524 | 375 | 24 | 42 | 18 | 924 | 1336 | 588 | 24 | 512 |

Table S6 continued

|  | **UCPH** | | | | | | | | | | | | **KCL** | | | | |
| --- | --- | --- | --- | --- | --- | --- | --- | --- | --- | --- | --- | --- | --- | --- | --- | --- | --- |
|  | **Replicate 1** | | | | | | **Replicate 2** | | | | | |  | | | | |
| **Locus** | **40 ng** | **10 ng** | **2 ng** | **0.5 ng** | **0.25 ng** | **0.125 ng** | **40 ng** | **10 ng** | **2 ng** | **0.5 ng** | **0.25 ng** | **0.125 ng** | **10 ng** | **2 ng** | **0.5 ng** | **0.25 ng** | **0.125 ng** |
| rs7148809 | 897 | 623 | 275 | 43 | 30 | 12 | 913 | 360 | 278 | 62 | 8 | 7 | 610 | 1533 | 676 | 55 | 0 |
| rs718501 | 673 | 498 | 318 | 50 | 21 | 7 | 808 | 378 | 214 | 28 | 2 | 4 | 537 | 801 | 531 | 10 | 5 |
| rs7198400 | 1238 | 994 | 632 | 195 | 106 | 3 | 1428 | 615 | 457 | 60 | 49 | 5 | 1138 | 2111 | 1028 | 221 | 0 |
| rs7252391 | 1022 | 678 | 288 | 73 | 126 | 13 | 1175 | 460 | 305 | 21 | 31 | 5 | 1117 | 1345 | 1585 | 1077 | 2 |
| rs7354930 | 1076 | 755 | 567 | 154 | 170 | 18 | 1442 | 547 | 270 | 38 | 10 | 13 | 1086 | 1752 | 1511 | 298 | 914 |
| rs7570971 | 1104 | 655 | 293 | 14 | 44 | 3 | 1128 | 439 | 223 | 33 | 36 | 16 | 529 | 788 | 284 | 329 | 370 |
| rs7594173 | 950 | 716 | 648 | 38 | 131 | 7 | 1192 | 510 | 521 | 35 | 18 | 20 | 633 | 1037 | 745 | 116 | 357 |
| rs7803075 | 1048 | 746 | 365 | 42 | 92 | 11 | 1196 | 461 | 389 | 59 | 63 | 2 | 948 | 1324 | 1078 | 196 | 21 |
| rs7814782 | 1110 | 784 | 308 | 70 | 77 | 9 | 1267 | 499 | 307 | 36 | 15 | 47 | 678 | 1278 | 923 | 211 | 149 |
| rs7816786 | 910 | 634 | 264 | 40 | 106 | 31 | 956 | 407 | 281 | 29 | 42 | 6 | 681 | 1556 | 1011 | 229 | 121 |
| rs7872110 | 876 | 668 | 278 | 58 | 86 | 0 | 1133 | 447 | 262 | 51 | 8 | 3 | 688 | 784 | 532 | 123 | 8 |
| rs7921384 | 1006 | 775 | 437 | 117 | 95 | 12 | 1143 | 419 | 436 | 45 | 7 | 58 | 759 | 1637 | 558 | 79 | 308 |
| rs7922818 | 1036 | 709 | 482 | 71 | 41 | 4 | 1009 | 417 | 317 | 33 | 1 | 4 | 727 | 1169 | 775 | 73 | 254 |
| rs7947221 | 899 | 879 | 384 | 123 | 80 | 1 | 1104 | 492 | 371 | 75 | 39 | 37 | 670 | 971 | 1106 | 6 | 284 |
| rs7975017 | 842 | 705 | 399 | 28 | 112 | 13 | 979 | 465 | 313 | 36 | 44 | 23 | 468 | 1105 | 782 | 197 | 6 |
| rs7981142 | 1168 | 902 | 527 | 107 | 89 | 0 | 1362 | 600 | 389 | 25 | 28 | 15 | 994 | 1208 | 954 | 145 | 351 |
| rs8027443 | 1125 | 816 | 296 | 113 | 53 | 5 | 1398 | 511 | 366 | 12 | 14 | 5 | 996 | 1154 | 918 | 174 | 236 |
| rs8064588 | 946 | 737 | 347 | 67 | 37 | 3 | 1150 | 411 | 243 | 35 | 4 | 40 | 504 | 711 | 355 | 124 | 102 |
| rs848461 | 1146 | 993 | 523 | 217 | 36 | 0 | 1266 | 541 | 340 | 53 | 1 | 68 | 962 | 1721 | 1051 | 119 | 450 |
| rs875001 | 993 | 830 | 489 | 48 | 35 | 4 | 1095 | 498 | 509 | 61 | 2 | 98 | 911 | 1290 | 1594 | 288 | 102 |
| rs896401 | 1087 | 856 | 407 | 21 | 2 | 2 | 1113 | 449 | 238 | 53 | 28 | 0 | 685 | 1610 | 786 | 208 | 0 |
| rs927140 | 1152 | 847 | 380 | 149 | 119 | 15 | 1238 | 514 | 370 | 26 | 40 | 37 | 990 | 1128 | 1100 | 174 | 92 |
| rs9315120 | 1086 | 843 | 381 | 136 | 57 | 8 | 1332 | 508 | 338 | 53 | 68 | 45 | 981 | 1281 | 1179 | 160 | 385 |
| rs932206 | 1211 | 778 | 516 | 103 | 129 | 1 | 1379 | 539 | 269 | 60 | 59 | 1 | 1127 | 1696 | 1591 | 140 | 2 |
| rs9467370 | 1067 | 695 | 422 | 81 | 88 | 11 | 1190 | 525 | 305 | 54 | 53 | 13 | 782 | 782 | 590 | 72 | 220 |
| rs947178 | 1126 | 802 | 453 | 78 | 51 | 25 | 1419 | 613 | 357 | 16 | 82 | 2 | 986 | 1399 | 1327 | 73 | 189 |

Table S6 continued

|  | **UCPH** | | | | | | | | | | | | **KCL** | | | | |
| --- | --- | --- | --- | --- | --- | --- | --- | --- | --- | --- | --- | --- | --- | --- | --- | --- | --- |
|  | **Replicate 1** | | | | | | **Replicate 2** | | | | | |  | | | | |
| **Locus** | **40 ng** | **10 ng** | **2 ng** | **0.5 ng** | **0.25 ng** | **0.125 ng** | **40 ng** | **10 ng** | **2 ng** | **0.5 ng** | **0.25 ng** | **0.125 ng** | **10 ng** | **2 ng** | **0.5 ng** | **0.25 ng** | **0.125 ng** |
| rs9479657 | 1193 | 1023 | 536 | 109 | 88 | 8 | 1291 | 558 | 320 | 65 | 27 | 67 | 1304 | 1775 | 1091 | 270 | 146 |
| rs9487258 | 933 | 615 | 500 | 116 | 78 | 1 | 992 | 440 | 320 | 22 | 0 | 46 | 936 | 1744 | 1022 | 114 | 207 |
| rs9493012 | 912 | 836 | 302 | 49 | 93 | 4 | 999 | 380 | 422 | 19 | 19 | 20 | 369 | 826 | 563 | 262 | 75 |
| rs9522149 | 1093 | 555 | 424 | 131 | 37 | 0 | 1207 | 449 | 295 | 120 | 11 | 45 | 938 | 1300 | 832 | 2 | 191 |
| rs964307 | 774 | 635 | 184 | 43 | 9 | 4 | 890 | 374 | 255 | 20 | 1 | 2 | 265 | 612 | 644 | 17 | 1 |
| rs9817359 | 1097 | 827 | 385 | 55 | 19 | 0 | 1103 | 474 | 276 | 38 | 24 | 41 | 876 | 1632 | 1653 | 363 | 0 |
| rs984038 | 1158 | 735 | 357 | 120 | 2 | 1 | 1227 | 491 | 223 | 19 | 14 | 1 | 885 | 1130 | 701 | 484 | 82 |
| rs9877982 | 1138 | 887 | 605 | 124 | 98 | 4 | 1299 | 641 | 492 | 64 | 14 | 3 | 1042 | 1617 | 688 | 235 | 328 |
| rs9899480 | 1243 | 851 | 604 | 69 | 109 | 10 | 1398 | 639 | 387 | 65 | 35 | 7 | 973 | 1464 | 1234 | 151 | 52 |
| rs9907597 | 1352 | 1148 | 529 | 137 | 73 | 11 | 1881 | 880 | 277 | 42 | 69 | 28 | 1339 | 1454 | 1980 | 41 | 101 |
